# The pathogenicity factor of DNA virus interacts with Arabidopsis deubiquitinases UBP12/13 to promote viral infection

**DOI:** 10.1371/journal.ppat.1014686

**Published:** 2026-10-15

**Authors:** Shun Li, Fangqin Yu, Yi Zhang, Siqi Liu, Hui-Wen Wu, Nam-Hai Chua, Shulin Deng

**Affiliations:** 1 Guangdong Provincial Key Laboratory of Applied Botany & Key Laboratory of National Forestry and Grassland Administration on Plant Conservation and Utilization in Southern China, South China Botanical Garden, Chinese Academy of Sciences, Guangzhou, China; 2 Plant Protection Research Institute, Guangdong Academy of Agricultural Sciences, Key Laboratory of Green Prevention and Control on Fruits and Vegetables in South China Ministry of Agriculture and Rural Affairs, Guangdong Provincial Key Laboratory of High Technology for Plant Protection, Guangzhou, People’s Republic of China; 3 University of Chinese Academy of Sciences, Beijing, China; 4 Temasek Life Sciences Laboratory, Singapore, Singapore; 5 School of Applied Science, Republic Polytechnic, Singapore, Singapore; Agriculture and Agri-Food Canada, CANADA

## Abstract

Plant DNA viruses, such as *Cauliflower mosaic virus* (CaMV) and *Cabbage leaf curl virus* (CaLCuV), are devastating pathogens that cause substantial yield losses in crops worldwide. These viruses assemble their genomes into minichromosomes with host histones. Histone post-translational modifications (PTMs) are important for viral replication and host resistance. In this study, we demonstrate that the CaMV pathogenicity factor P6 interacts with Arabidopsis deubiquitinases UBP12 and UBP13, which are typically negative regulators of plant immunity. Using affinity purification–mass spectrometry (AP-MS), we identified UBP12/13 as prominent P6 interactors and confirmed their nuclear association. CaMV infection and P6 overexpression increased UBP12 abundance, suggesting the latter promotes viral infection. By contrast, *ubp12/13* mutants exhibited enhanced resistance, characterized by reduced accumulation of viral DNA and transcripts. Chromatin immunoprecipitation (ChIP)-qPCR showed that the monoubiquitinated H2A (H2Aub) and H3K27me3 modifications on the CaMV 35S promoter, but not the 19S promoter, were significantly elevated in *ubp12/13* mutants compared with wild-type plants. Furthermore, UBP12/13 are also targeted by AC2, a transcriptional activator from CaLCuV, indicating a conserved strategy among plant DNA viruses. Our findings support a working model in which viral pathogenicity factors hijack UBP12/13, which in turn may alter H2Aub homeostasis on viral minichromosomes to relieve epigenetic silencing and promote infection. Together, these findings point to a previously unrecognized route by which plant DNA viruses may influence host chromatin-associated pathways and identify UBP12/13 as potential targets for engineering virus-resistant crops.

## Author summary

Plant DNA viruses, including double-stranded DNA viruses (caulimoviruses) and single-stranded DNA viruses (geminiviruses), are critical crop pathogens worldwide. Upon infection, viral genomes are packaged with host histones to form minichromosomes, and histone post-translational modifications (PTMs) on these minichromosomes play a critical role in regulating viral replication and gene expression. In this study, we identified that Arabidopsis deubiquitinases UBP12 and UBP13 are recruited by the P6 protein of CaMV might influence the epigenetic state of the 35S promoter in viral minichromosomes. We confirmed that P6 directly interacts with UBP12/13 in the nucleus, and CaMV infection and P6 overexpression specifically enhance UBP12 protein abundance. We found that *ubp12/13* mutants showed enhanced resistance to CaMV and had reduced accumulation of viral DNA. In addition, the pathogenicity factor AC2 of CaLCuV also hijacks UBP12/13 to facilitate DNA virus infection. Collectively, our results illustrate that UBP12/13 act as conserved host factors exploited by viral pathogenicity proteins to promote viral infection.

## Introduction

Viral pathogens cause significant damage to crop growth and production. In response to virus infection, plants mount multiple defense mechanisms to block virus multiplication and propagation. Conversely, viruses produce proteins that interact with various host proteins to suppress and counteract host defense responses and facilitate viral replication and gene expression. DNA viruses, such as caulimoviruses and geminiviruses, use host histones to assemble their DNA intermediates into minichromosomes, which support viral replication and gene expression [1,2]. Similar to eukaryotes, post-translational histone modifications (PTMs) are crucial for gene expression of viral minichromosomes [3]. Therefore, DNA viruses recruit host histone modification machinery by interacting with plant proteins.

*Cauliflower mosaic virus* (CaMV), a typical member of the caulimoviruses (family *Caulimoviridae*), is classified among the pararetroviruses (PRVs). This virus infects most *Brassicaceae* plants as well as several *Solanaceae* plants leading to significant yield losses in broccoli, cauliflower, cabbage, and turnip [4]. Upon infection, the CaMV genome is packaged by host histones to form minichromosomes [1,5]. The viral genome is transcribed into two mRNAs: the polycistronic 35S RNA, which encodes five proteins (P1, P2, P3, P4, and P5), and the 19S RNA, which produces the essential P6 protein [5–7]. P6, a multi-functional protein, impacts various stages of CaMV-host interactions. Initially identified as a major component of viral inclusion bodies, P6 influences viral replication and assembly [7,8]. It induces symptoms such as chlorosis and mottling, facilitates cell-to-cell virus movement [9,10] and triggers systemic cell death and hypersensitive response (HR) in *Nicotiana* species [11,12]. P6 functions as a nucleocytoplasmic shuttle protein with two nuclear import signals and one nuclear export signal [13,14] and acts as an RNA silencing suppressor in the nucleus [13,15,16]. Given that the CaMV genome exists as minichromosomes, it is important to investigate whether nuclear-localized P6 contributes to the transcriptional regulation of viral genes.

Previous studies have reported that histone PTMs also occur on viral minichromosomes, which influence viral gene expression. Raja *et al* demonstrated that histone H3 methylation is associated with methylated viral DNA in geminivirus-infected plants, and histone H3 methylation levels on viral DNA are reduced in methylation-deficient mutants that display severe disease symptoms [17]. The Rep protein of the geminivirus, *Chilli leaf curl virus* (ChiLCV), recruits NbUBC2 and NbHUB1 to monoubiquitinate H2B (H2Bub), subsequently increasing H3K4me3 levels on the ChiLCV minichromosome, which eventually enhance viral gene expression [3]. Whereas monoubiquitination of H2B is an activation mark for transcription, monoubiquitinated H2A (H2Aub) generally represses gene transcription. Although histone H2A monoubiquitination (H2Aub) is a crucial histone PTM that regulates host gene transcription and its role in regulating gene expression from viral minichromosomes remains unclear.

Ubiquitination can regulate functions of substrate proteins by altering their activity and mediating their degradation. Ubiquitination is a reversible PTM that can be reversed by deubiquitinating enzymes (DUBs) [18]. In Arabidopsis, DUBs are classified into five subfamilies, of which the ubiquitin-specific proteases (UBPs), possess the largest members of the DUB family [18]. UBPs are multifunctional proteins that contribute to plant immunity and other physiological processes. In Arabidopsis, UBP12 and UBP13 participate in diverse pathways, such as light perception, photoperiod regulation, hormonal signalling, growth and development and responses to environmental stress [19–23].

UBP12/13 are known to be involved in plant immunity against virulent *Pseudomonas syringae* pv. tomato (*Pst* DC3000) mediated by salicylic acid (SA) signaling [24]. Both UBP12 and its *Solanaceous* orthologue NtUBP12 are required to suppress Cf-9-dependent plant cell death [25]. Additionally, UBP12 and UBP13 exhibit DUB activity towards histone H2Aub and influence gene expression by altering H2Aub and H3K27me3 levels on target genes in Arabidopsis [26,27].

Here, we provide several lines of evidence that viral pathogenicity proteins associate with cellular deubiquitinases involved in histone PTMs and may influence the chromatin state of viral minichromosomes during infection. First, we showed that the CaMV P6 protein interacted with Arabidopsis deubiquitinases UBP12/13, and that deficiency of UBP12/13 in Arabidopsis delayed CaMV infection and reduced the accumulation of viral DNA and transcripts. We then observed that UBP12 protein levels increased in *P6* overexpression lines and CaMV-infected plants. Furthermore, we found that UBP12/13 affected H2Aub and H3K27me3 deposition on CaMV minichromosomes. Finally, we showed that UBP12/13 are common host proteins associated with pathogenicity factors of not only CaMV but also the geminivirus *Cabbage leaf curl virus* (CaLCuV), suggesting a possible conserved link between viral pathogenicity factors and host chromatin-associated pathways.

## Results

### CaMV P6 interacts with Arabidopsis deubiquitinases UBP12/13

To elucidate the role of P6 in facilitating CaMV infection, we first identified P6-interacting proteins in extracts of transgenic plants over-expressing FLAG-P6 using affinity purification coupled with mass spectrometry (AP-MS). Consistent with earlier reports, *FLAG-P6*-overexpressing plants exhibited CaMV-like symptoms including chlorosis, serrated leaf margins, mosaic patterns, stunting and delayed flowering (Fig 1A and 1B). Compared with those from *35S:FLAG-EGFP* control plants, 36 proteins were significantly enriched in *FLAG-P6* immunoprecipitates (S1 Table). Notably, most of these proteins were associated with chromatin modification and nuclear regulation, providing key clues for subsequent functional validation. This chromatin-associated set included histone modification enzymes and chaperones such as HD2C, UBP12, UBP13, PIE1, and PAH2, among which UBP12 and UBP13 ranked highest (Fig 1C, S1 Table). GO molecular function analysis of these 36 P6-interacting proteins identified RNA binding (29.67%), protein binding (17.58%), structural molecule activity (9.89%), and catalytic activity (9.89%) as the dominant functional categories (S1 Fig). Additionally, 11 60S ribosomal proteins formed the largest interactor group (Fig 1C, S1 Table), consistent with the reported translational transactivation activity of P6. A second group of eight proteins was involved in RNA processing and degradation, including NUC-L1, NUC-L2, VCS, and the decapping complex component PAT1 (S1 Table).

**Fig 1:**
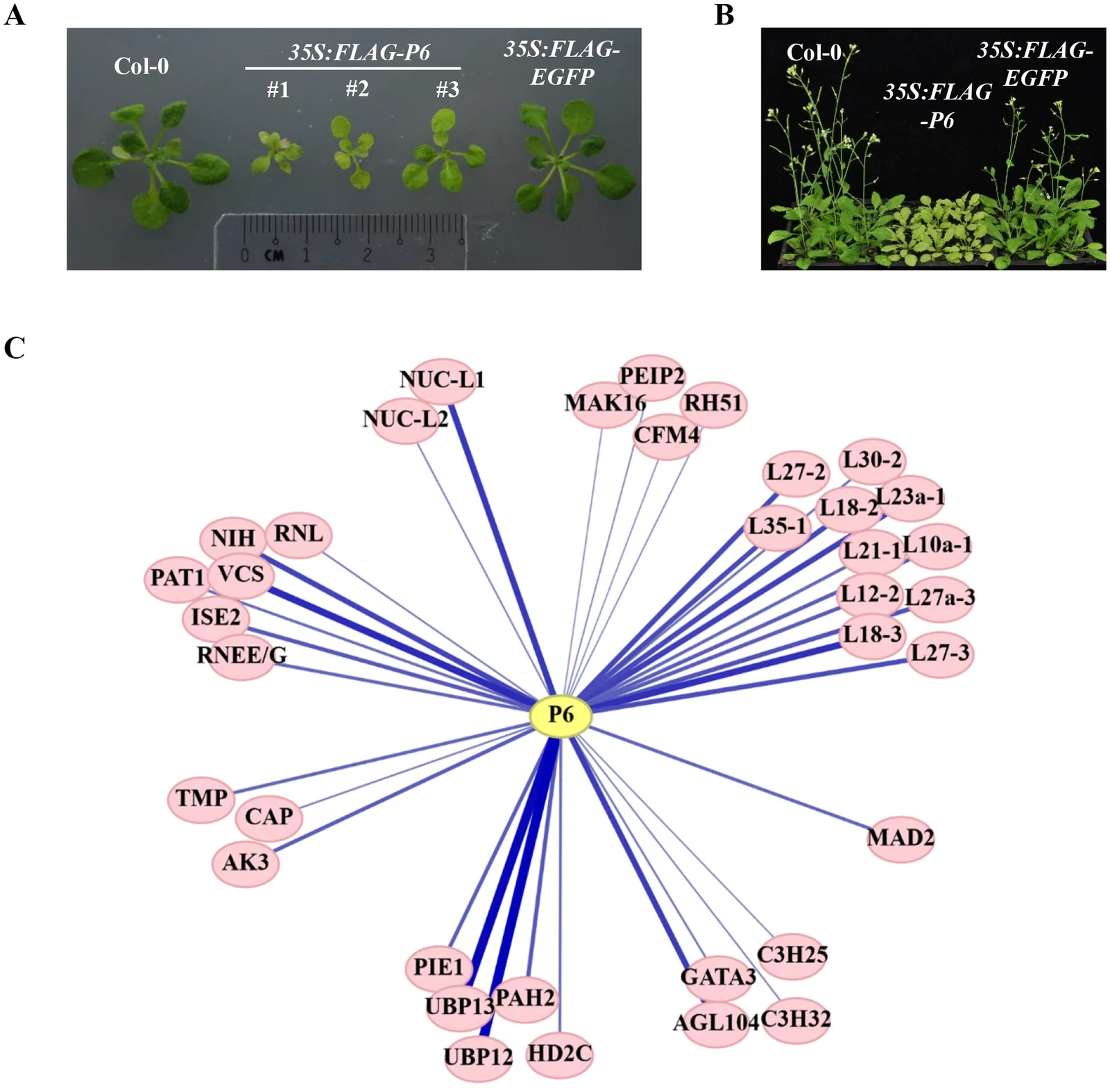
Interacting proteins of *Cauliflower mosaic virus* (CaMV) P6 in Arabidopsis. (A, B) Arabidopsis overexpression lines of P6 show CaMV symptom-like phenotypes. (C) An interaction map of P6 and its identified interacting proteins. The thickness of the line shows the strength of interaction. Transgenic Arabidopsis plants expressing *35S:FLAG-P6* were used to analyze binding proteins co-immunoprecipitated with FLAG-P6. Proteins were identified by mass spectrometry after proteolytic digestion. Plants expressing *35S:FLAG-EGFP* were used as negative control.

Given their top abundance in the AP-MS dataset, we next focused on the deubiquitinases UBP12/13. To validate the AP-MS results, we performed yeast two-hybrid (Y2H) assays to confirm the interaction of P6 with UBP12/13 as well as with other viral proteins (P1, P2, P3, P4, and P5). Y2H assays confirmed that UBP12/13 interacted exclusively with P6 but not with other viral proteins (Fig 2A). Both UBP12 and UBP13 contain a protein interaction domain known as MATH (meprin and TRAF homology) at the N-terminal region and a protease domain called USP (ubiquitin-specific protease) at the C-terminal region [28]. To map the interaction domain, we split UBP12/13 into two fragments: the N-terminal MATH domain and the C-terminal region (S2A Fig). Y2H assays showed that the MATH domain was required for the interaction of P6 with UBP12/13 (S2B Fig). We further examined the *in vitro* interaction by pull-down assays: purified MBP-P6 was precipitated by GST-UBP12 and GST-UBP13 but not by GST alone (Fig 2B). The in vitro interaction between P6 and UBP12/13 was confirmed in vivo by co-immunoprecipitation assays (Co-IP) using transgenic Arabidopsis co-expressing *YFP-P6* and *UBP12/13-FLAG* in the *ubp12–1* or *ubp13–2* mutant backgrounds (Fig 2C). To confirm that the interaction was not restricted to Arabidopsis, we conducted Co-IP assays in *Nicotiana benthamiana* and observed similar results (S3 Fig).

**Fig 2:**
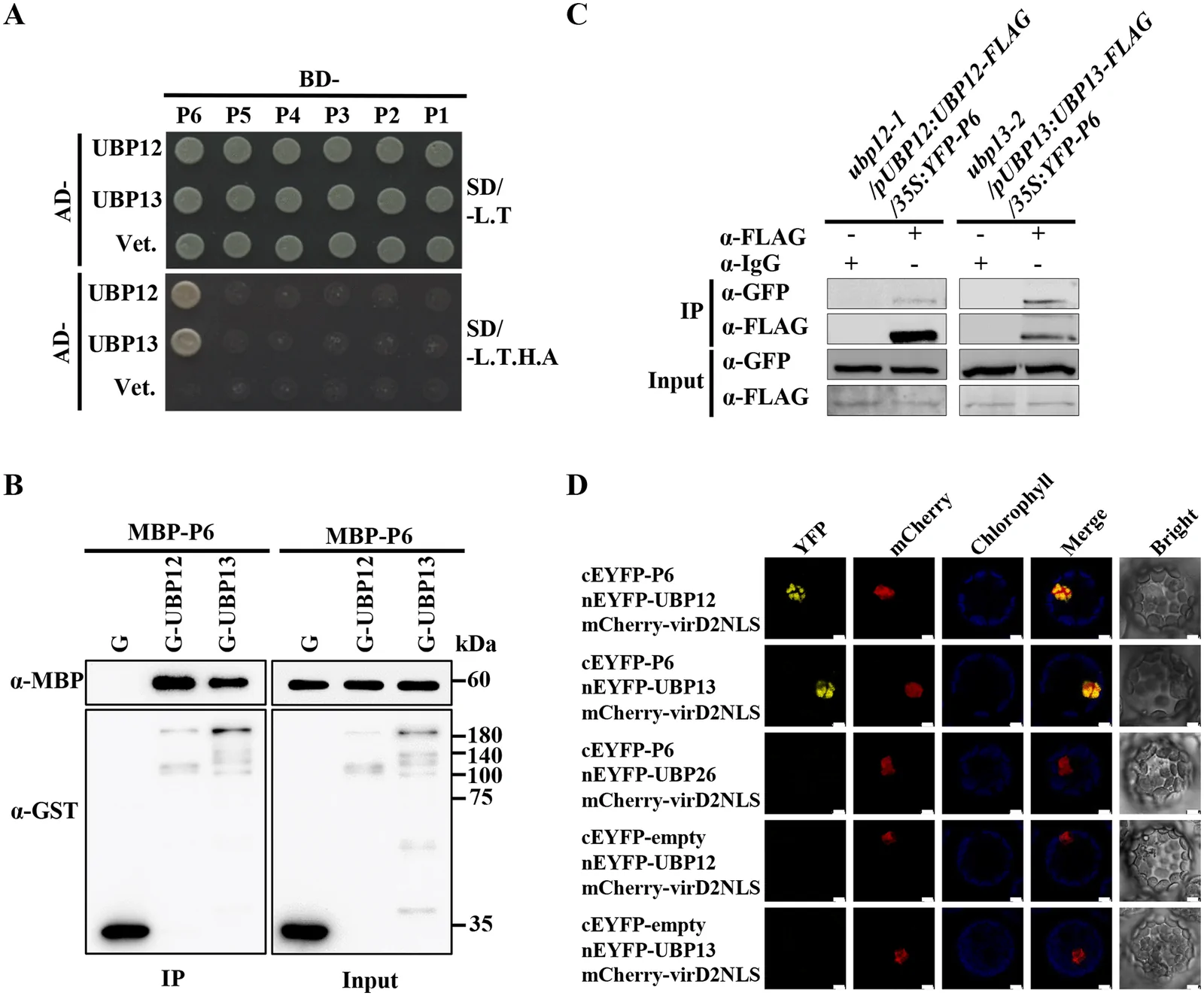
CaMV P6 interacts with Arabidopsis deubiquitinases UBP12/13. (A) Yeast two-hybrid (Y2H) assay of UBP12/13 and CaMV proteins. Yeast co-transformants grown on nonselective media (-Leu/-Trp) and selective media (-Leu/-Trp/-His/-Ade) were shown. Vet.: empty vector. SD/-L.T indicates Leu and Trp drop-out medium. SD/-L.T.H.A indicates Leu, Trp, His and Ade drop-out medium. (B) *In vitro* pull-down assay shows a direct interaction between GST-UBP12/13 and MBP-P6. GST-UBP12, GST-UBP13 and GST (negative control) were used as baits. MBP-P6 were used as target protein. G: GST. (C) Co-immunoprecipitation (Co-IP) assay for detecting the interaction between UBP12/13 and P6 using transgenic Arabidopsis co-expressing P6 and UBP12/13. Extracts were immunoprecipitated with anti-FLAG antibody and anti-IgG antibody (negative control). Immunoprecipitates were then analyzed by immunoblotting assay using anti-GFP and anti-FLAG antibody. Input controls were shown. (D) Bimolecular fluorescence complementation (BiFC) assays between UBP12/13 and P6 in Arabidopsis protoplasts. Confocal imaging was performed at 12 h post inoculation. UBP12/13 and P6 were fused to nEYFP and cEYFP, respectively. mCherry-VirD2NLS was co-expressed as a nuclear marker. Bar, 5 μm.

Furthermore, bimolecular fluorescence complementation (BiFC) assays in Arabidopsis protoplasts and *N. benthamiana* epidermal cells showed reconstituted YFP fluorescence in the nucleus when cEYFP-P6 was co-expressed with nEYFP-UBP12 or nEYFP-UBP13, whereas no signal was detected with nEYFP-UBP26, a close homolog of UBP12 and UBP13 (Figs 2D and S2C).

### Arabidopsis *ubp12/13* mutants exhibit delayed CaMV-mediated disease development

To investigate the roles of UBP12 and UBP13 in antiviral defense and their interaction with CaMV, we analyzed corresponding mutants and overexpressing lines in Arabidopsis. The *ubp12–2* mutant (GABI_742C10), which displayed severe developmental defects including dwarf stature and altered leaf morphology [29], was therefore excluded from further analysis. Following inoculation, disease progression was monitored by scoring symptom severity using a 1–5 scale based on chlorosis, vein-yellowing, rugosity and leaf bunching symptoms (S2 Table). Wild-type (WT) plants developed moderate symptoms (severity grade 3) whereas the *ubp12/13* mutants exhibited milder symptoms (grade 2) and showed an approximate 0.5–1.5-day delay in reaching the half-maximal disease severity (Fig 3A and 3B). Consistent with symptom severity, viral DNA accumulation was significantly reduced in *ubp12/13* mutants compared to Col-0 WT plants (Fig 3C). By contrast, *UBP12/13* overexpression lines displayed more severe symptoms (grades 4–5) and reached half-maximal disease severity earlier than WT (S4A–S4C Figs). To further assess viral replication, transcript levels of *P1*, *P3*, *P5*, and *P6* were quantified by qPCR. Transcript levels of these viral genes were significantly reduced in *ubp12/13* mutants compared to WT (Fig 3D) whereas their accumulation was elevated in *UBP12/13* overexpression lines (S4D and S4E Figs). In addition, we detected continuous expression of UBP12/13 over the course of CaMV infection, where UBP12 protein abundance displayed an upward trend at 9–15 dpi (S5 Fig). Together, these findings indicate that *UBP12* and *UBP13* function redundantly to promote CaMV infection and disease development in Arabidopsis and that deficiency of *UBP12/13* enhances host resistance to CaMV.

**Fig 3:**
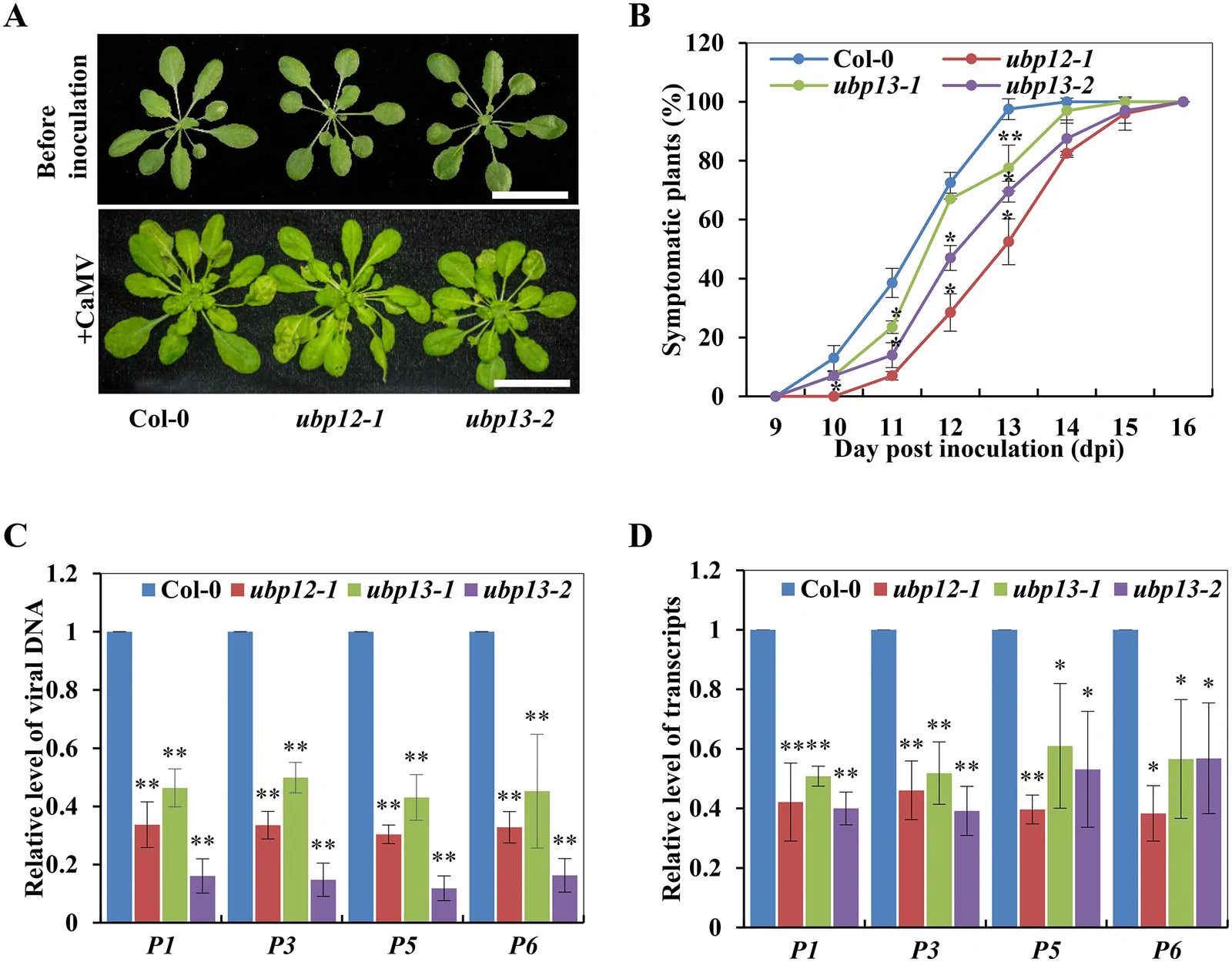
UBP12/13 function redundantly as negative regulators of defense to CaMV infection. (A) Phenotypes of wild-type (WT) and *ubp12/13* mutants prior to CaMV inoculation and at 16 days post-inoculation (dpi). Bar, 3 cm. (B) Infectivity assay of CaMV on WT and *ubp12/13* mutants. Percentages of symptomatic plants at different days after inoculation were shown. (C) Relative accumulation of CaMV DNA (P1, P3, P5, P6) in infected wild-type plants and *ubp12/13* mutants at 14 dpi by qPCR assay. (D) Transcript levels of *P1*, *P3*, *P5*, *P6* in WT and *ubp12/13* mutants at 14 dpi with CaMV. The statistical significance between the mean ± standard deviation (SD) values were calculated by performing student’s *t*-test (**P < 0.01, *P < 0.05), n = 3 biological replicates.

To test whether UBP12 deubiquitinase activity is associated with its pro-viral function during CaMV infection, we evaluated viral DNA accumulation in plants expressing wild-type UBP12 or its catalytic-dead C/S variant. We performed PVX-based transient expression in C24-background Arabidopsis. Following PVX-driven expression of wild-type UBP12, the C/S mutant, or empty vector (EV) control, plants were inoculated with CaMV. All experimental groups shared the same PVX backbone to maintain comparable baseline conditions. Immunoblot analysis confirmed that protein abundance of PVX-driven UBP12 and UBP12-C/S was comparable (S6A Fig). Consistent with symptom severity, viral DNA accumulation measured by qPCR was significantly higher in PVX-UBP12 plants than in PVX-EV plants (S6B and S6C Figs). In contrast, the C/S mutant did not show a similar increase in viral DNA accumulation, and viral DNA levels even lower than those observed in the EV control (S6B and S6C Figs). The high level of UBP12-C/S mutant driven by PVX vector conferred to a dominant negative mutant phenotype. We next generated stable Arabidopsis UBP12-OX and UBP12-C/S-OX transgenic lines in the WT background. Immunoblotting also verified similar protein levels of UBP12-OX and UBP12-C/S-OX in these stable transgenic plants (S6D Fig). In agreement with our transient-assay data, CaMV DNA accumulation was elevated in UBP12-OX plants, whereas this enhancement was not observed in UBP12-C/S-OX plants (S6E and S6F Figs). Collectively, these results suggest that UBP12 deubiquitinase activity is required for its pro-viral role during CaMV infection.

### CaMV infection and overexpression of *P6* increase UBP12 protein abundance

UBP12 and UBP13 have been reported to function as deubiquitinating enzymes that regulate protein stability, such as UBP13 stabilizing the receptor protein RGFR1 in root meristem growth [30]. To investigate whether UBP12/13 proteins influence P6 stability, we quantified P6 abundance in Arabidopsis lines co-expressing *35S:MYC-P6* and *XVE:UBP12-FLAG* or *XVE:UBP13-FLAG*, with or without β-estradiol induction. Comparable P6 protein levels were detected in induced and non-induced plants suggesting that UBP12/13 do not regulate P6 protein stability (Fig 4A). Next, we examined whether CaMV infection alters *UBP12/13* expression. Transcript and protein levels were monitored in complementation lines (*pUBQ12:UBP12-FLAG* in *ubp12–1* and *pUBQ13:UBP13-FLAG* in *ubp13–2*) following CaMV inoculation. RT-PCR analysis revealed no significant changes in *UBP12* or *UBP13* transcript levels (Fig 4B). By contrast, UBP12-FLAG levels increased in infected plants, whereas UBP13-FLAG levels remained unchanged (Fig 4B). Consistently, transient expression of P6 in *N. benthamiana* specifically elevated UBP12-FLAG levels without affecting UBP13-FLAG levels (Fig 4C). To confirm these findings in Arabidopsis, we analyzed UBP12 and UBP13 protein levels in complementation lines with or without P6 induction. As shown in Fig 4D, P6 induction significantly increased UBP12-FLAG abundance in *ubp12–1/pUBP12:UBP12-FLAG/XVE:MYC-P6* transgenic plants, whereas UBP13-FLAG levels remained unchanged in *ubp13–2/pUBP13:UBP13-FLAG/XVE:MYC-P6* plants. Collectively, these results show that both CaMV infection and *P6* overexpression specifically enhance UBP12 protein abundance without affecting UBP13 levels.

**Fig 4:**
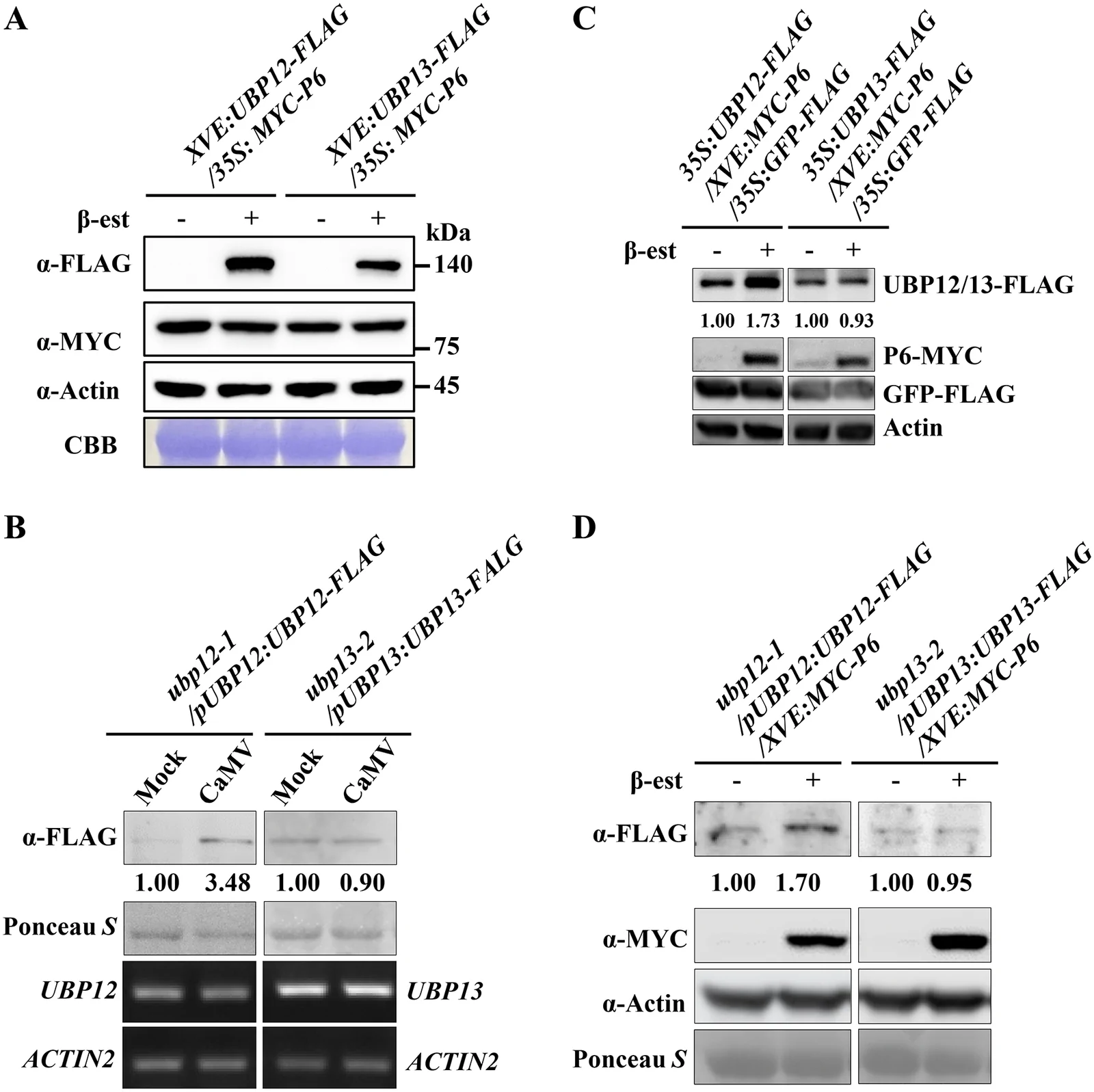
CaMV infection or overexpression of P6 increases UBP12 protein abundance. (A) Detection of MYC-P6 stability in Arabidopsis co-expressing *35S: MYC-P6* and *XVE:UBP12-FLAG* or *XVE:UBP13-FLAG*. Two-week-old seedlings were incubated in liquid MS medium with or without β-estradiol (β-est). UBP12/13-FLAG and MYC-P6 were detected by immunoblot using anti-FLAG and anti-MYC antibodies, respectively. Actin immunoblotting and Coomassie Brilliant Blue (CBB) staining were used as loading controls. (B) UBP12 and UBP13 transcripts and protein accumulation in *UBP12* complementation line and *UBP13* complementation line inoculated with or without CaMV at 14 dpi. UBP12/13-FLAG were detected by anti-FLAG antibody. The bottom two panels show transcript levels of *UBP12*/*UBP13* and *ACTIN2* (control) determined by RT-PCR. (C) Detection of UBP12 and UBP13 accumulation in *Nicotiana benthamiana*. *Agrobacterium* strains containing *XVE:MYC-P6* and *35S:UBP12-FLAG* or *35S:UBP13-FLAG* were co-infiltrated into *N. benthamiana*. *Agrobacterium* containing *35S:GFP-FLAG* was also co-infiltrated as control. Proteins were detected by immunoblot using anti-FLAG, anti-MYC, anti-GFP and anti-Actin (loading control) antibodies. (D) UBP12 and UBP13 protein levels in UBP12 or UBP13 complementation lines co-expressing *XVE:MYC-P6*. Two-week-old seedlings were incubated in liquid MS medium with or without β-estradiol (25 μM) for 1 d. UBP12/13-FLAG and MYC-P6 were detected by immunoblot using anti-FLAG and anti-MYC antibodies, respectively. Actin immunoblotting and Ponceau *S*-stained membrane (RUBISCO large subunit) were used as loading controls. Numbers below the blots indicate relative protein abundance (B, C, D).

### UBP12/13 affect H2Aub and H3K27me3 enrichment on CaMV minichromosomes

Because UBP12/13 are known to deubiquitinate monoubiquitinated H2A (H2Aub) [26], we hypothesized that their interaction with P6 could modulate the epigenetic state of CaMV minichromosomes during infection. Therefore, we performed chromatin immunoprecipitation (ChIP) assays using antibodies against H2Aub, H3K27me3, and H3K4me3, followed by qPCR analysis of five regions (p1–p5) spanning the CaMV promoters (Fig 5A). Compared with inoculated Col-0 WT plants, H2Aub levels were significantly increased on the CaMV 35S promoter in *ubp12–1* and *ubp13–1* mutants (Fig 5A and 5B). Because H2Aub deposition is frequently linked to Polycomb complexes recruitment and subsequent H3K27me3 accumulation [27], we next assessed H3K27me3 levels. ChIP analysis revealed a significant increase in H3K27me3 modification on viral promoter regions in both *ubp12/13* mutants (Fig 5C). By contrast, H3K4me3, a histone mark linked to transcriptional activation [3], showed no significant difference between mutants and Col-0 WT plants (Fig 5D). Collectively, these ChIP-qPCR data indicate that loss of UBP12/13 is associated with increased levels of the repressive marks H2Aub and H3K27me3 on CaMV viral minichromosomes, whereas the activating histone mark H3K4me3 remains largely unchanged.

**Fig 5:**
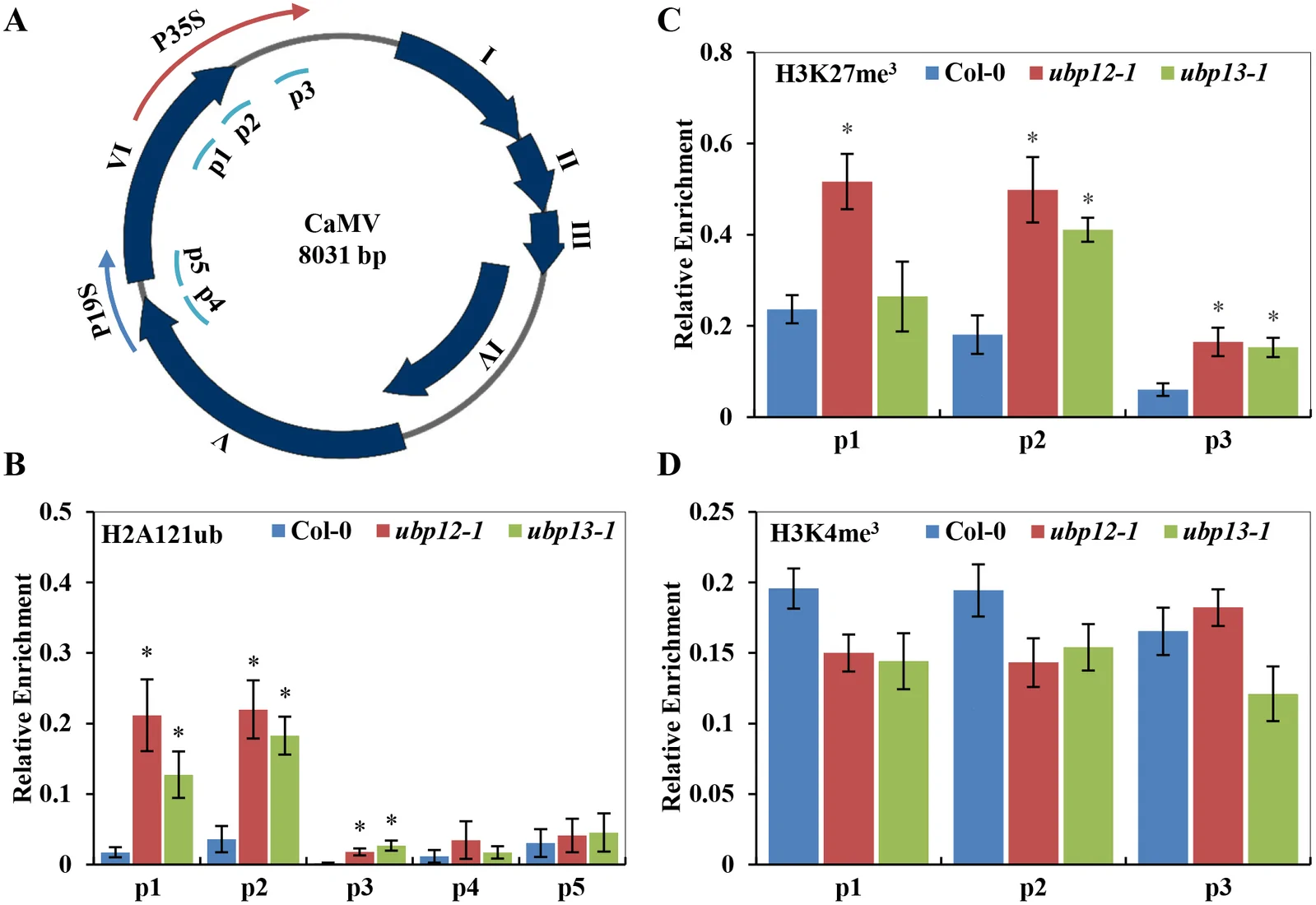
UBP12/13 influence H2Aub and H3K27me3 enrichment on the CaMV minichromosome. (A) Schematic representation of the CaMV genome. The 19S and 35S promoter regions are indicated by blue and red arrows, respectively. Fragments p1–p5 were selected for Chromatin Immunoprecipitation qPCR (ChIP-qPCR). (B–D) ChIP-qPCR analysis of H2Aub (B), H3K27me3 (C), and H3K4me3 (D) enrichment at the CaMV promoter in Col-0, *ubp12-1*, and *ubp13-1* plants at 21 dpi with CaMV. Enrichment levels of immunoprecipitated DNA using specific antibodies were normalized to input DNA. *ACTIN7* was used as an internal control across samples. Data represents mean ± SD, with n = 3 biological replicates. Statistical significance was determined using Student’s *t*-test (**P < 0.01, *P < 0.05).

### UBP12/13 are hijacked by the pathogenicity factor AC2 of CaLCuV to facilitate infection

Because both CaMV and geminiviruses form chromatinized minichromosomes in the host nucleus [1,25,31], we hypothesized that UBP12/13 might regulate chromatin modifications in a conserved manner across plant DNA viruses. To examine this hypothesis, we extended our analysis to CaLCuV, a geminivirus that infects Arabidopsis and is known to form histone-associated viral minichromosomes [17].

To assess the role of UBP12/13 in CaLCuV infection, we inoculated *ubp12/13* mutants and Col-0 WT plants and monitored disease progression. Compared with Col-0 WT plants, *ubp12/13* mutants exhibited milder symptoms and reduced disease severity (Fig 6A). Consistent with these observations, qPCR results revealed significantly lower levels of viral DNA and transcripts of the viral genes, *BL1*, *AC2*, and *AC4* in t*ubp12/13* plants (Figs 6B and 6C).

**Fig 6:**
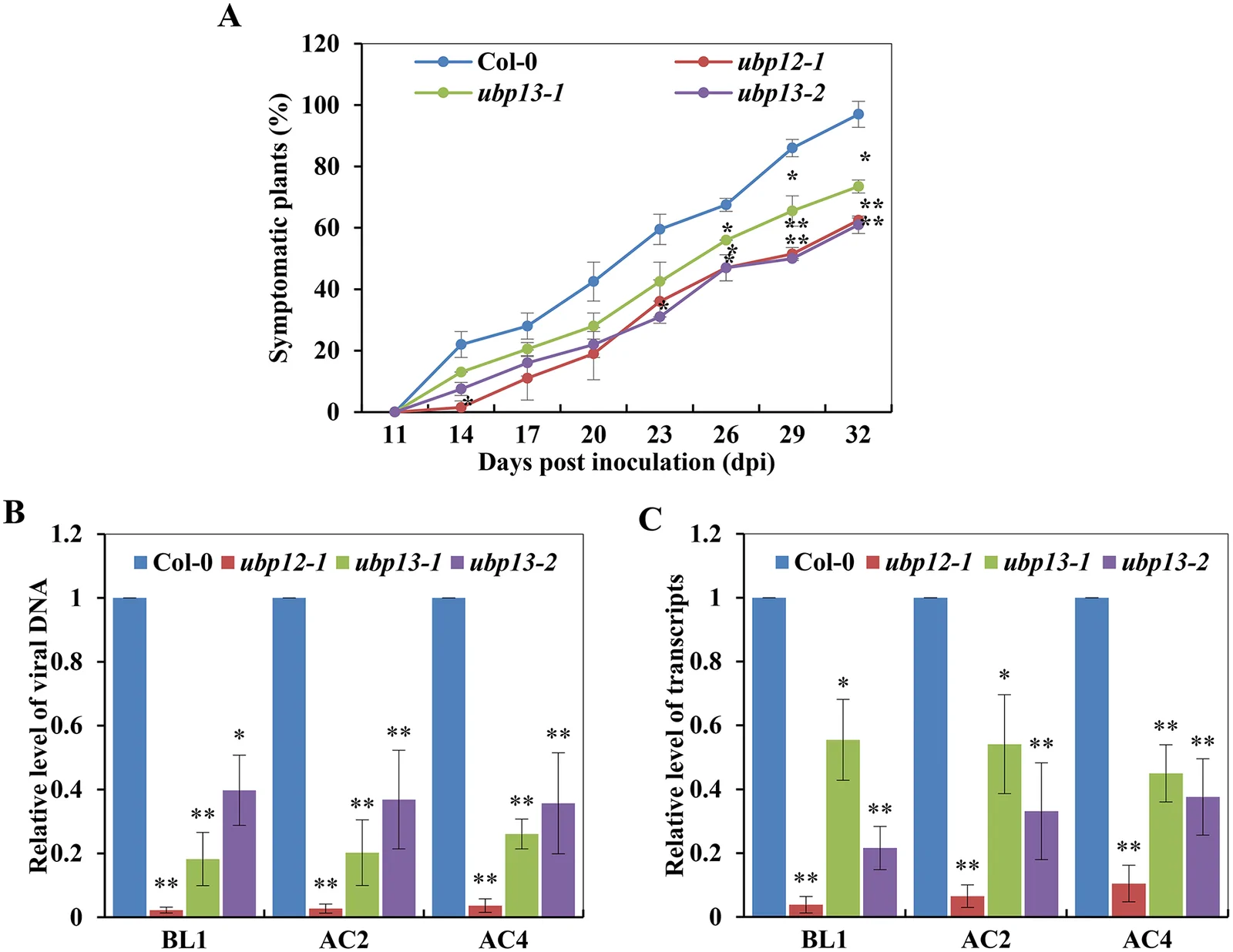
UBP12/13 function redundantly as negative regulators of defense to CaLCuV infection. (A) Infectivity assay of CaLCuV on Col-0 and *ubp12/13* mutants. Percentages of symptomatic plants at different days after inoculation are shown. (B) CaLCuV DNA (BL1, AC2, AC4) accumulation levels in infected WT and *ubp12/13* mutants at 28 dpi by qPCR assay. (C) Expression analysis of BL1, AC2, AC4 transcripts level in WT and *ubp12/13* mutants at 28 dpi inoculated with CaLCuV. The statistical significance between the mean ± SD values were calculated by performing students’ *t*-test (**P < 0.01, *P < 0.05), n = 3.

CaLCuV encodes AC2, a pathogenicity factor that suppresses RNA silencing, modulates host metabolism and regulates transcription [32–35], similar to the multifunctional role of CaMV P6. Given these parallel properties, we investigated whether AC2 also targets UBP12/13. We performed *in vitro* pull-down assay using MBP-tagged AC2 and GST-tagged UBP12/13. MBP-AC2 was efficiently captured by GST-UBP12 and GST-UBP13, but not by GST alone (Fig 7A). We next confirmed this interaction *in vivo* using co-immunoprecipitation assays. AC2 co-immunoprecipitated with UBP12 and UBP13 in Arabidopsis lines expressing these proteins (Figs 7B and S7). Furthermore, BiFC assay in *N. benthamiana* epidermal cells showed that AC2-cEYFP interacted with UBP12/UBP13-nEYFP in the nucleus, while no fluorescence signal was observed in empty vector controls (Fig 7C). Consistent with the reduced susceptibility of *ubp12/13* mutants to CaLCuV infection (Fig 6), these results indicate that the CaLCuV pathogenicity factor AC2 physically associates with UBP12 and UBP13 during infection. Whether this interaction contributes to chromatin-based regulation of the geminiviral minichromosome remains to be determined.

**Fig 7:**
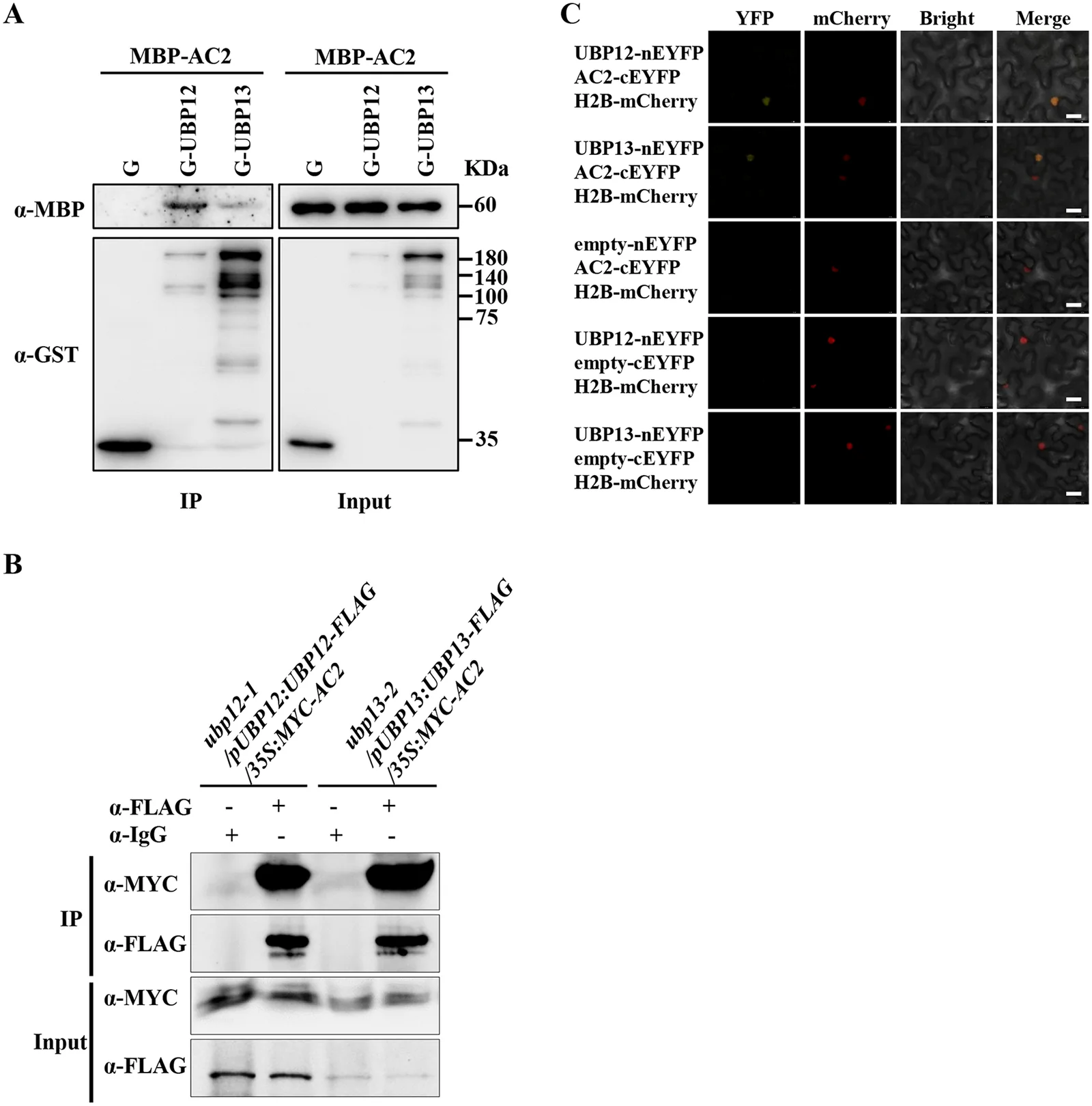
CaLCuV AC2 interacts with UBP12/13. (A) *In vitro* pull-down assay shows a direct interaction between GST-UBP12/13 and MBP-AC2. GST-UBP12, GST-UBP13 and GST (control) were used as baits. MBP-AC2 was used as the target protein. G: GST. (B) Co-immunoprecipitation (Co-IP) assay for detecting the interaction between AC2 and UBP12/13 using the transgenic Arabidopsis co-expressing AC2 and UBP12/13. Extracts were immunoprecipitated with anti-FLAG antibody and anti-IgG antibody. Immunoprecipitates were then analyzed by immunoblotting assay using anti-MYC and anti-FLAG antibody. Input proteins were shown. (C) BiFC assays between UBP12/13 and AC2 in *N. benthamiana*. Confocal images were taken 2 days after infiltration. UBP12/13 and AC2 were respectively fused to nEYFP and cEYFP. H2B-mCherry was as a nuclear protein marker. Bar, 20 μm.

## Discussion

Plant DNA viruses that replicate in the nucleus must overcome host chromatin-based defense mechanisms that restrict transcription from viral genomes. Increasing evidence indicates that plant DNA viruses actively manipulate host epigenetic pathways to counteract this barrier [17,36,37]. In this study, we identify the Arabidopsis deubiquitinases UBP12 and UBP13 as host factors targeted by plant DNA viruses and provide evidence that viral association with host deubiquitination pathways may contribute to infection.

DNA viruses such as CaMV and CaLCuV utilize host histones to package their genomes into minichromosomes [1,25,31]. To suppress the viral proliferation, hosts employ epigenetic control of chromatin through various histone PTMs to repress gene expression from the viral minichromosome [17]. As a counter-defense, viruses recruit host chromatin-modifying enzymes to overcome this repression epigenetically. For example, the geminivirus ChiLCV Rep protein recruits NbHUB1 and NbUBC2 to monoubiquitinate histone H2B on viral minichromosomes, thereby enhancing H3K4me3 deposition and promoting viral gene expression [3]. In this context, our data favour a working model whereby CaMV may remodel the chromatin landscape of its minichromosomal genome by co-opting host deubiquitinases (Fig 8). Engagement of viral pathogenicity effectors with UBP12 and UBP13 is hypothesized to contribute to diminished repressive histone modifications on viral DNA, potentially promoting transcription from viral minichromosomes during infection. Although our ChIP-qPCR analyses clearly show elevated H2Aub and H3K27me3 at CaMV minichromosomes in *ubp12/13* mutants, the exact molecular mechanisms through which viral proteins recruit or functionally modulate these host chromatin regulators remain to be further defined. UBP12/13 share homology with human deubiquitinase USP7/HAUSP, with approximately 34% amino acid sequence identity [29]. Notably, USP7 overexpression or disruption of its viral interactor ICP0 promotes viral mRNA and protein accumulation [38], suggesting a conserved role for DUBs in viral infection. Together, these observations suggest that host deubiquitination activity may be associated with a chromatin environment that is more permissive for CaMV gene expression.

**Fig 8:**
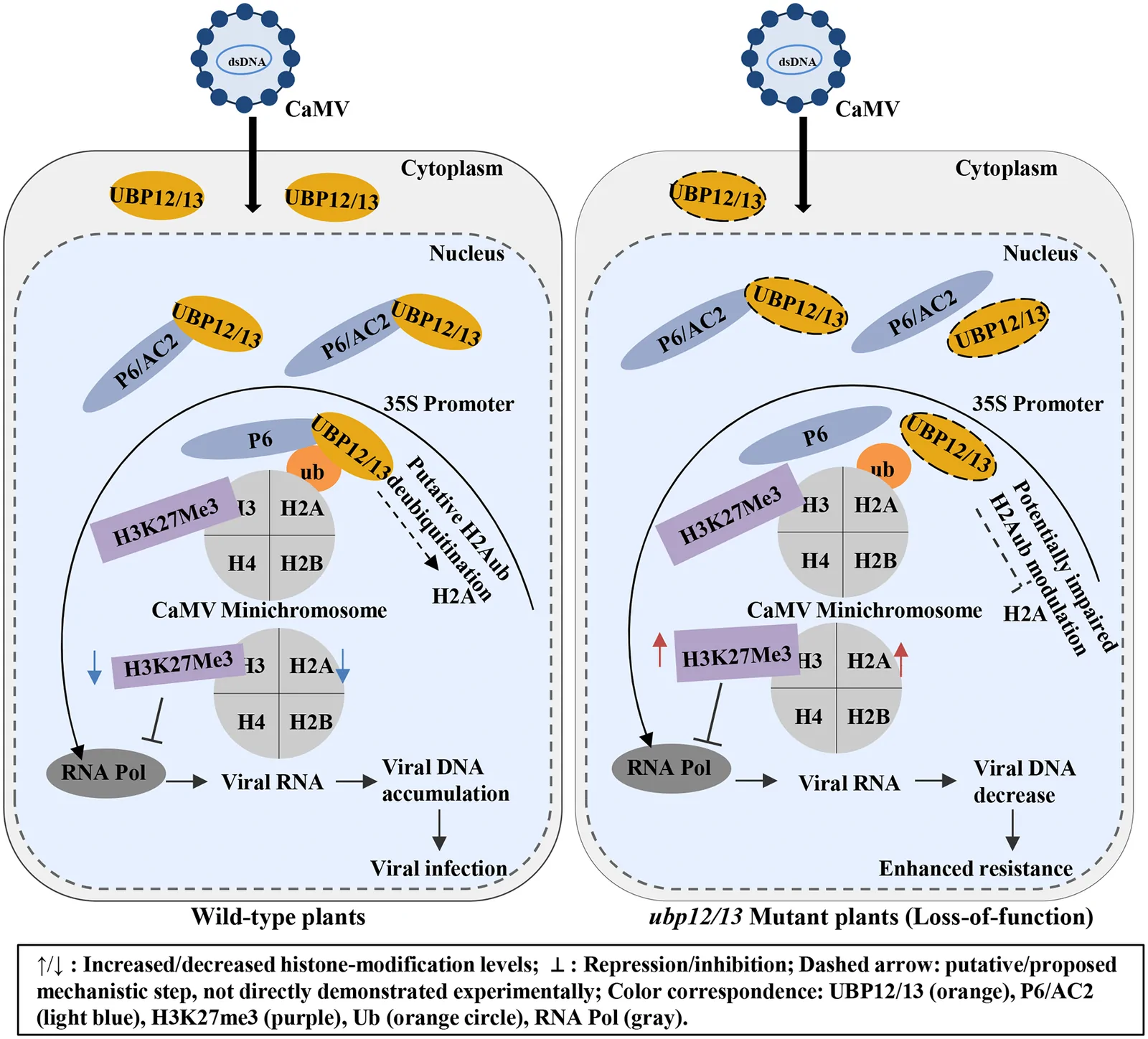
Proposed working model illustrating how CaMV P6 exploits UBP12/13 to alleviate host epigenetic silencing and promote viral infection, representing a conserved strategy among plant-infecting DNA viruses. In wild-type plants (left panel), UBP12/13 localize to both the cytoplasm and nucleus and interact with CaMV P6 or the geminivirus effector AC2 in the nucleus. P6-associated UBP12/13 may contribute to changes in H2Aub levels at the 35S promoter of CaMV viral minichromosomes. This association appears promoter-specific, as no detectable changes in histone modifications were observed at the 19S promoter. In wild-type plants, lower H2Aub coincides with lower H3K27me3 at the 35S promoter; conversely, both repressive marks accumulate in *ubp12/13* mutants. The potential causal relationship between H2Aub and H3K27me3 dynamics. Reduced H3K27me3 is associated with diminished transcriptional repression, which may create a chromatin environment more permissive for viral transcription and viral RNA/DNA accumulation during CaMV infection. In *ubp12/13* loss-of-function mutant plants (right panel), elevated H2Aub and H3K27me3 persist at the 35S promoter, correlating with sustained viral transcriptional repression. This correlates with restricted viral transcript and DNA accumulation and enhanced plant resistance to CaMV. Symbol key: Overlapping ovals indicate protein-protein interactions; upward/downward arrows indicate increased/decreased histone modification levels; ┴ indicates repression or inhibition; dashed arrows denote putative or proposed mechanistic steps not yet directly demonstrated experimentally; dashed ovals indicate loss-of-function or catalytically inactive proteins. Color code: UBP12/13 (orange), P6/AC2 (light blue), H3K27me3 (purple), ubiquitin (orange circle), RNA Pol (gray).

Beyond chromatin regulation, UBP12/13 have been implicated in protecting proteins from 26S proteasome-mediated degradation. For instance, UBP12/13 stabilize ORE1 during nitrogen-deficiency–induced senescence [20] and UBP12 regulates the stability of the RGF1 receptor during root meristem maintenance [30]. In our system, UBP12/13 did not alter P6 stability in our system; instead, CaMV infection and P6 overexpression were associated with increased UBP12 abundance. A similar increase in the UBP12/13 homolog USP7 has been reported during HIV-1 infection, although the underlying mechanism remains unclear [39]. In Arabidopsis, *UBP12/13* transcripts are upregulated during CaLCuV infection at 12 dpi [40,41] but not in response to AC2 overexpression, suggesting virus-specific regulation of UBP12/13 abundance. The mechanisms regulating UBP12 and UBP13 protein levels remain unknown and warrant further investigation. Based on previous findings that CaMV P6 activates TOR kinase [42,43] and that UBP12 interacts with TOR complex scaffold protein AtRaptor1B [44], we hypothesize that UBP12/13 turnover may involve autophagy-mediated degradation, which could be suppressed when P6 activates TOR signaling. Conversely, UBP12/13 might influence TOR stability or activity, forming a potential feedback loop. These hypotheses remain speculative and require future experimental validation.

Beyond its well-documented function in suppressing RNA silencing, the nuclear functions of CaMV P6 remain incompletely characterized [13]. Our results are consistent with a possible nuclear function for CaMV P6, whereby its association with UBP12/13 may facilitate viral transcription. This observation is consistent with previously reported roles of UBP12/13 in regulating H2Aub and H3K27me3 at Polycomb-regulated host loci [26,27]. Similarly, the geminivirus ChiLCV employs its replication initiator protein (Rep) to recruit NbUBC2 and NbHUB1, which drive histone H2B monoubiquitination and subsequent elevation of H3K4me3 on viral minichromosomes to boost viral gene expression [3]. Collectively, these findings favour a working model in which diverse viral effectors may exploit host chromatin-modifying machinery to derepress viral transcription, potentially representing a broader strategy to counteract host epigenetic silencing (Fig 8). Notably, our ChIP-qPCR data reveal coordinated changes of H2Aub and H3K27me3 at the CaMV 35S promoter in *ubp12/13* mutants. Nevertheless, these observations reflect a correlative association rather than proven causality. Whether altered H2Aub directly drives changes in H3K27me3 at viral minichromosomes, or vice-versa, remains untested. Therefore, the functional link between these two epigenetic marks at viral promoters requires further experimental validation.

UBP12/13 are also known to negatively regulate plant immunity. UBP12/13 deubiquitinate and stabilize the salicylic acid (SA) receptor NPR3, thereby suppressing SA-dependent immune responses [24]. In tobacco, UBP12/13 similarly function as negative regulators of immunity against bacterial pathogen *Pst* DC3000 [45]. During CaMV infection, SA-responsive genes including *PR-1*, *PR-2*, and *PR-5* are expressed at low levels prior to 8 dpi, whereas viral transcripts accumulate rapidly between 5 and 8 dpi [46]. These observations raise the possibility that the enhanced resistance of *ubp12/13* mutants reflects combined effects of altered immune signalling and chromatin-based restriction of viral transcription. These mechanisms are not mutually exclusive and may act in parallel to restrict viral proliferation.

The interaction of CaLCuV AC2 with UBP12/13 suggests that geminiviruses may co-opt host deubiquitinases to influence transcriptional outcomes, potentially via chromatin-associated mechanisms. AC2 functions as a transcriptional activator with weak dsDNA binding and can activate the coat protein gene promoter in mesophyll cells through interaction with PEAPOD2 [34,35]. These considerations raise the possibility that AC2 may indirectly targets promoters, potentially enabling geminiviruses to modulate host and viral gene expression from minichromosomes. We therefore propose that CaLCuV AC2 interacts with UBP12/13 during infection, potentially contributing to viral pathogenicity through mechanisms that remain to be fully defined. Consistent with chromatin-based counter-defense strategies in geminiviruse, C2/AC2 from *Tomato golden mosaic virus* (TGMV) and CaLCuV interact with the H3K9me2 methyltransferase SUVH4/KYP, preventing its association with viral minichromosomes and facilitating infection [37].

An outstanding question is whether histone modifiers are actively recruited to, or displaced from, viral minichromosomes; and how the timing and directionality of these events are regulated during infection. In summary, this study identifies host deubiquitinases as key nodes exploited by CaMV and potentially other plant DNA viruses, and highlights chromatin regulation as a critical interface in plant-virus interactions. By integrating protein-protein interaction, genetic, and chromatin-based analyses, our findings provide a framework for understanding how viral pathogens may influence host epigenetic machinery during infection.

## Methods

### Plant growth and inoculation

Wild-type (Col-0; WT) *Arabidopsis thaliana* and previously characterized null mutant alleles *ubp12–1* (GABI_244E11), *ubp12–2* (GABI_742C10), *ubp13–1* (SALK_128312) and *ubp13–2* (SALK_024054) were used in this study. Arabidopsis plants were grown in a controlled growth chamber under an 8-h light/ 16-h dark cycle at 23°C. CaMV inoculum was prepared by homogenizing 0.1 g of Arabidopsis leaves infected with pCa122 (carrying CM1841 viral DNA) in 1 mL of sterile 0.05 M phosphate buffer (pH 7.2) [47,48]. For inoculation, five basal rosette leaves of three-week-old Arabidopsis were gently rubbed with a cotton swab soaked in the CaMV virion solution. Mock-inoculated plants were treated identically using sterile phosphate buffer instead of virus solution. For assays involving β-estradiol-inducible lines, plants were sprayed with 25 µM β-estradiol supplemented with 0.1% (v/v) Tween-20 at 1 d prior to CaMV inoculation. Subsequent treatments with 10 µM β-estradiol (0.1% Tween-20) were applied by foliar spraying every 3 d after inoculation. For CaMV and CaLCuV infection assays, thirty-two plants per genotype were used in each of three independent biological replicates.

### Plasmid construction, transient expression, and virus inoculation

UBP12-Flag and the catalytic-dead UBP12-Flag (UBP12-C/S) were cloned into the PGR107 (PVX) vector. *Agrobacterium*-mediated transient expression in *N. benthamiana* was performed as described previously [49,50]. *Agrobacterium* cultures were adjusted to an OD_600_ of 0.5 for *N. benthamiana* infiltration. For inoculation of three-week-old C24 Arabidopsis, the swab-inoculation method was performed as described by Brosseau et al. [50]. Briefly, sap was extracted from *N. benthamiana* plants infected with PVX-EV, PVX-UBP12, or PVX-UBP12-C/S by grinding tissue in 0.1 M phosphate buffer (pH 7.0; 2 mL/g tissue). Each PVX-derived sap was mixed separately with CaMV inoculum; buffer was used for mock control. Sap from uninfected tobacco tissue was prepared in parallel to serve as the mock inoculation control (2 mL/g healthy tissue).

### Afﬁnity puriﬁcation-mass spectrometry

Leaf tissues from homozygous *35S:FLAG-P6* transgenic Arabidopsis lines, as well as from WT and *35S:FLAG-EGFP* control plants, were used for AP-MS. WT plants served as a control for non-specific binding to the matrix, whereas *35S:FLAG-EGFP* plants were used to exclude FLAG-tag-dependent interactors. For immunoprecipitation, total protein was extracted from 2 mL of tissue powder using an ice-cold extraction buffer A (50 mM Tris-HCl pH 7.5, 150 mM NaCl, 0.5% (v/v) Triton X-100, 100 mM sodium orthovanadate, 0.005% (w/v) leupeptin, 1 × EDTA-free protease inhibitor cocktail (Roche, Cat. No. 11873580001), and one PhosStop (Roche, Cat. No. 04906845001) per 10 mL). The extract was incubated on ice for 15 min and then diluted 1:1 with extraction buffer B (buffer A lacking Triton X-100). After centrifugation at 2900 *× g* for 10 min at 4°C, the supernatants were filtered through a 0.45-μm nylon membrane. Pre-cleared lysates were incubated with 100 μL Protein A agarose for 1 h at 4°C with rotation, then transferred to pre-washed anti-FLAG M2 affinity gel, FLAG-Trap A (ChromoTek) and incubated for an additional 2 h at 4°C. The beads were then washed with washing buffer A (50 mM Tris-HCl, 100 mM NaCl, 0.05% Triton X-100) three times and washing buffer B (washing buffer A without Triton X-100) twice. Samples on the beads were eluted by boiling with SDS loading buffer and then reduced at 50°C for 15 min by adding 30 µL of 100 mM dithiothreitol (DTT). For alkylation, samples were incubated for 30 min at room temperature in the dark with 1:10 2-iodoacetamide (1 M). After overnight digestion by trypsin (Promega, Cat. No. V5113) at 37°C, samples dissolved in 0.1% trifluoroacetic acid (TFA, pH 2.0) were loaded onto C18 SepPak columns pre-equilibrated with 0.1% TFA, before the pump was switched on. After rinsing three times with 0.1% TFA, peptides were eluted by 800 μL of 60% acetonitrile in 0.1% TFA and eluants were dried to completeness in a SpeedVac concentrator (Thermo Fisher Scientific). Mass spectrometric analysis was carried out by using a Nano LC 1000 liquid chromatography system with a reversed-phase C18 column (ReproSil, Dr. Maisch, Ammerbuch, Germany). Peptides were dissolved in 40 μL of 0.2% TFA/5% acetonitrile and analyzed using an Amazon ETD mass spectrometer (Bruker Daltronik).

### Yeast two-hybrid assays

Yeast two-hybrid (Y2H) assays were carried out following the Yeast Protocols Handbook (Clontech). Full-length coding sequences (CDSs) of UBP12 and UBP13, together with those of CaMV proteins (P1, P2, P3, P4, P5, P6), were cloned into pGADT7 or pGBKT7. All constructs, including the corresponding empty vectors, were transformed into yeast strain *AH109*. Transformants were first selected on non-selective medium (-Leu/-Trp) and then assayed on selective medium (-Leu/-Trp/-His/-Ade or -Leu/-Trp/-His).

### Pull-down assays

Full-length CDSs of *UBP12* and *UBP13* were amplified and cloned into pGEX-4T-1, while the *P6* and *AC2* coding sequences were inserted into pMAL-c2X. The resulting plasmids were transformed into *E. coli Rosetta*. Expression was induced by adding IPTG to 0.2 mM final concentration, followed by incubation at 28°C for 4 h with shaking. MBP beads (NEB, Cat. No. E8021S) and GST beads (GE Healthcare, Cat. No. 17-0756-01) were used to purify the MBP-fusion proteins and GST-fusion proteins, respectively. For pull-down assays, GST-2HA, GST-UBP12–2HA, or GST-UBP13–2HA immobilized on GST beads were incubated with MBP-P6-2FLAG or MBP-AC2–2FLAG in pull-down buffer (PBS, 1% Triton X-100) at 4°C for 2 h. After 6 washes, bound proteins were eluted and detected by immunoblotting with anti-MBP (Santa Cruz, Cat. No. sc-809), anti-FLAG (Sigma, Cat. No. SAB4200071), or anti-HA (AlpaLifeBio, Cat. No. KTSM1315) antibodies.

### Co-immunoprecipitation assay

Co-immunoprecipitation assays were performed as described [24]. Total proteins from two-week-old transgenic Arabidopsis plants were incubated with Protein A/G agarose beads (Millipore, Cat. No. IP10) pre-conjugated to anti-FLAG antibody (Millipore, Cat. No. F7425) or pre-immune IgG in IP buffer (50 mM Tris-HCl pH 7.5, 100 mM NaCl, 10 mM MgCl_2_, 0.1% Nonidet P-40, 1 mM PMSF, 10% glycerol, 1 mM DTT, 50 μM MG132, and 1 × protease inhibitor cocktail) at 4°C for 2 h. After six washes with IP buffer, immunoprecipitated proteins were analyzed by immunoblotting using anti-GFP (Santa Cruz, Cat. No. sc-9996), anti-MYC (Santa Cruz, Cat. No. 9E10), or anti-FLAG (Sigma, Cat. No. SAB4200071) antibodies.

### DNA and RNA extraction and quantification

Total RNA was isolated with Trizol reagent, followed by DNase I treatment (QiagencDNA was synthesized using the GoScript Reverse transcription kit (Promega, Code No. A2709). Genomic DNA was extracted with cetyltrimethylammonium bromide (CTAB) buffer. Real-time PCR was performed using SYBR Premix Ex Taq (Takara, Cat. No. RR420A) and gene-specific primers on a LightCycler 480 instrument (Roche). All quantification experiments were carried out with three independent biological replicates and normalized with *ACTIN2* expression levels. Primers used in this assay are listed in S3 Table.

### Chromatin immunoprecipitation (ChIP)

Systemic leaves (2 g) displaying symptoms were harvested from Arabidopsis plants at 21 dpi after CaMV inoculation and used for chromatin immunoprecipitation (ChIP) following a published protocol with minor modifications [51]. After cross-linking, chromatin pellets were resuspended in nuclei lysis buffer, and chromosomal DNA was sonicated into fragments. Cleared supernatants were incubated with salmon-sperm DNA/Protein A agarose beads pre-conjugated to anti-H2Aub (Cell Signaling Technology, Cat. No. 8240), anti-H3K27me3 (Abcam, Cat. No. ab6002), or anti-H3K4me3 (Abcam, Cat. No. ab8580) antibodies. Purified DNA was analyzed by real-time PCR using *ACTIN7* as internal control. Primers used are listed in S4 Table.

### BiFC assay

DNA sequences encoding the tested proteins were cloned into pGreen-II binary vectors carrying nEYFP or cEYFP as described previously [52]. mCherry-VirD2NLS served as a nuclear marker. Plasmids were co-transformed into Arabidopsis mesophyll protoplasts, which were then cultured overnight. For BiFC in *N. benthamiana*, the constructs were individually introduced into *Agrobacterium tumefaciens* strain *GV3101*. Transient transformation of *N. benthamiana* leaves was performed as described previously [53]. YFP fluorescence was observed using confocal microscopy. H2B-mCherry served as a nuclear localization marker.

### Protein detection

Total proteins were extracted from Arabidopsis or transiently transformed *N. benthamiana* leaves using extraction buffer (50 mM Tris-HCl pH 7.5, 150 mM NaCl, 2 mM EDTA, 0.5% Triton X-100, 0.5 mM PMSF, 10% glycerol, 10 mM DTT, and 1 × protease inhibitor cocktail). Western blot analysis was performed using anti-MYC (Santa Cruz, Cat. No. 9E10), anti-FLAG (Sigma, Cat. No. SAB4200119), anti-GFP (Santa Cruz, Cat. No. SC-9996), and anti-Actin (Abbkine, Cat. No. A01050) antibodies.

## Supporting information

S1 Fig Functional categorization of 36 P6-interacting proteins according to Gene Ontology (GO) molecular function terms. The 36 candidate proteins were identified via affinity purification coupled with mass spectrometry (AP-MS), and their functional classification was conducted using the GO annotation tool available at https://v2.arabidopsis.org/tools/bulk/index.jsp, using the corresponding gene locus identifiers as input.
(TIF)

S2 Fig The MATH domain is required for UBP12/13 interaction with P6. (A) Schematic representation of UBP12 and UBP13 protein structures. The MATH domain, Cys-box, and His-box are indicated as circles or rectangles. (B) Yeast two-hybrid (Y2H) assay showing the UBP12/13 protein domains required for interaction with P6. Growth of yeast cells expressing UBP12/13 and P6 on non-selective medium (SD/-Leu/-Trp) and selective medium (SD/-Leu/-Trp/-His) is shown. “Vet.” indicates empty vector control. SD/-L.T refers to medium lacking Leu and Trp; SD/-L.T.H refers to medium lacking Leu, Trp, and His. (C) BiFC assays between UBP12/13 and AC2 in *N. benthamiana*. Confocal images were taken 2 days after infiltration. UBP12/13 and P6 were respectively fused to nEYFP and cEYFP. H2B-mCherry was as a nuclear protein marker. Bar, 20 μm.
(TIF)

S3 Fig UBP12/13 interact with P6 in *Nicotiana benthamiana.* Fusion protein UBP12/13-FLAG or GUS-FLAG were transiently co-expressed with MYC-P6 in *N. benthamiana*. MYC-P6 co-immunoprecipitated with UBP12-FLAG and UBP13-FLAG but not GUS-FLAG which used as a negative control. Fusion proteins were detected by immunoblotting using anti-FLAG and anti-MYC antibodies. Ponceau *S* staining was used as a loading control.
(TIF)

S4 Fig *UBP12/13* over-expression lines are sensitive to *Cauliflower mosaic virus* (CaMV). (A) Detection of UBP12/13 proteins in *UBP12/13* over-expression lines. Two-week-old seedlings of *XVE:UBP12-FLAG* lines and *XVE:UBP12-FLAG* lines were incubated in liquid MS medium with or without β-estradiol (25 μM) for 1 d. UBP12-FLAG and UBP13-FLAG proteins were extracted and detected using anti-FLAG antibody by immunoblots. Ponceau *S* staining was used as a loading control. (B) Phenotypes of wild-type plant and *UBP12/13* over-expression lines inoculated with CaMV at 16 dpi. Bar, 3 cm. (C) Infectivity assay of CaMV on wild-type plants and *UBP12/13* over-expression lines. Percentages of symptomatic plants at different days after inoculation are shown. Data points at each time point from 8 to 16 dpi represent mean ± SD. (D) CaMV DNA (P1, P3, P5, P6) accumulation level in infected wild-type plants and *UBP12/13* over-expression lines at 14 dpi by qPCR assay. (E) Expression analysis of *P1*, *P3*, *P5*, *P6* transcripts level in wild-type plants and *UBP12/13* over-expression lines at 14 dpi inoculated with CaMV. The statistical significance between the mean ± SD values were calculated by performing student’s *t*-test (**P < 0.01, *P < 0.05).
(TIF)

S5 Fig UBP12/13 exhibit sustained overexpression during CaMV infection, with UBP12 protein abundance gradually increasing from 9 to 15 days post-inoculation (dpi). Young leaves were harvested at 9, 12, and 15 dpi, and UBP12/13 protein accumulation dynamics were determined by Western blot. Actin immunoblotting and Coomassie Brilliant Blue (CBB) staining served as loading controls. Numbers below the blots indicate relative protein abundance.
(TIF)

S6 Fig The deubiquitinase activity of UBP12 is required for its pro-viral function during CaMV infection. (A) Immunoblot detection of UBP12 and UBP12-C/S protein levels in the PVX transient-expression system. CBB staining served as the loading control. (B) Plant phenotypes of PVX-EV, PVX-UBP12, and PVX-UBP12-C/S before CaMV inoculation and at 16 dpi. Bar, 2 cm. (C) Relative accumulation of CaMV DNA (P1, P3, P5, and P6) in PVX-EV, PVX-UBP12, and PVX-UBP12-C/S infected plants at 12 dpi, as measured by qPCR. (D) Immunoblot detection of UBP12 and UBP12-C/S protein levels in UBP12-OX and UBP12-C/S-OX stable Arabidopsis transgenic plants. CBB staining served as the loading control. (E) Phenotypes of WT, UBP12-OX, and UBP12-C/S-OX stable Arabidopsis transgenic plants before CaMV inoculation and at 20 dpi. Bar, 2 cm. (F) Relative accumulation of CaMV DNA (P1, P3, P5, P6) in infected WT, UBP12-OX, and UBP12-C/S-OX plants at 15 dpi, as measured by qPCR. Statistical significance was determined using Student’s *t*-test. Data are presented as mean ± SD (**P < 0.01, *P < 0.05).
(TIF)

S7 Fig UBP12/13 interact with AC2 in *Nicotiana benthamiana.* Fusion protein UBP12/13-FLAG or GUS-FLAG were transiently co-expressed with MYC-AC2 in *N. benthamiana*. MYC-AC2 co-immunoprecipitated with UBP12-FLAG and UBP13-FLAG but not GUS-FLAG which is used as a negative control. Fusion proteins were detected by immunoblotting using anti-FLAG and anti-MYC antibodies. CBB staining was used as loading control.
(TIF)

S1 Table P6 candidate interactors identified through IP-MS. (XLSX)

S2 Table Growth and disease development of mutants and overexpressing lines of UBP12/13 compared to the wild-type. (XLSX)

S3 Table Primers used PCR and RT-PCR analysis. (XLSX)

S4 Table Primers used for ChIP-PCR assay. (XLSX)

S1 Raw Gel Original images for blots and gels in article’s figures and supporting information files. (PDF)
