# Supplementary material for: The pathogenicity factor of DNA virus interacts with Arabidopsis deubiquitinases UBP12/13 to promote viral infection
Source: PLoS Pathog. 2026 Oct 15;22(10):e1014686. doi: 10.1371/journal.ppat.1014686 (PMC13690193; doi:10.1371/journal.ppat.1014686)
Supplement: Supplementary file 1 [file ppat.1014686.s012.pdf]

Fig 2B_raw_image

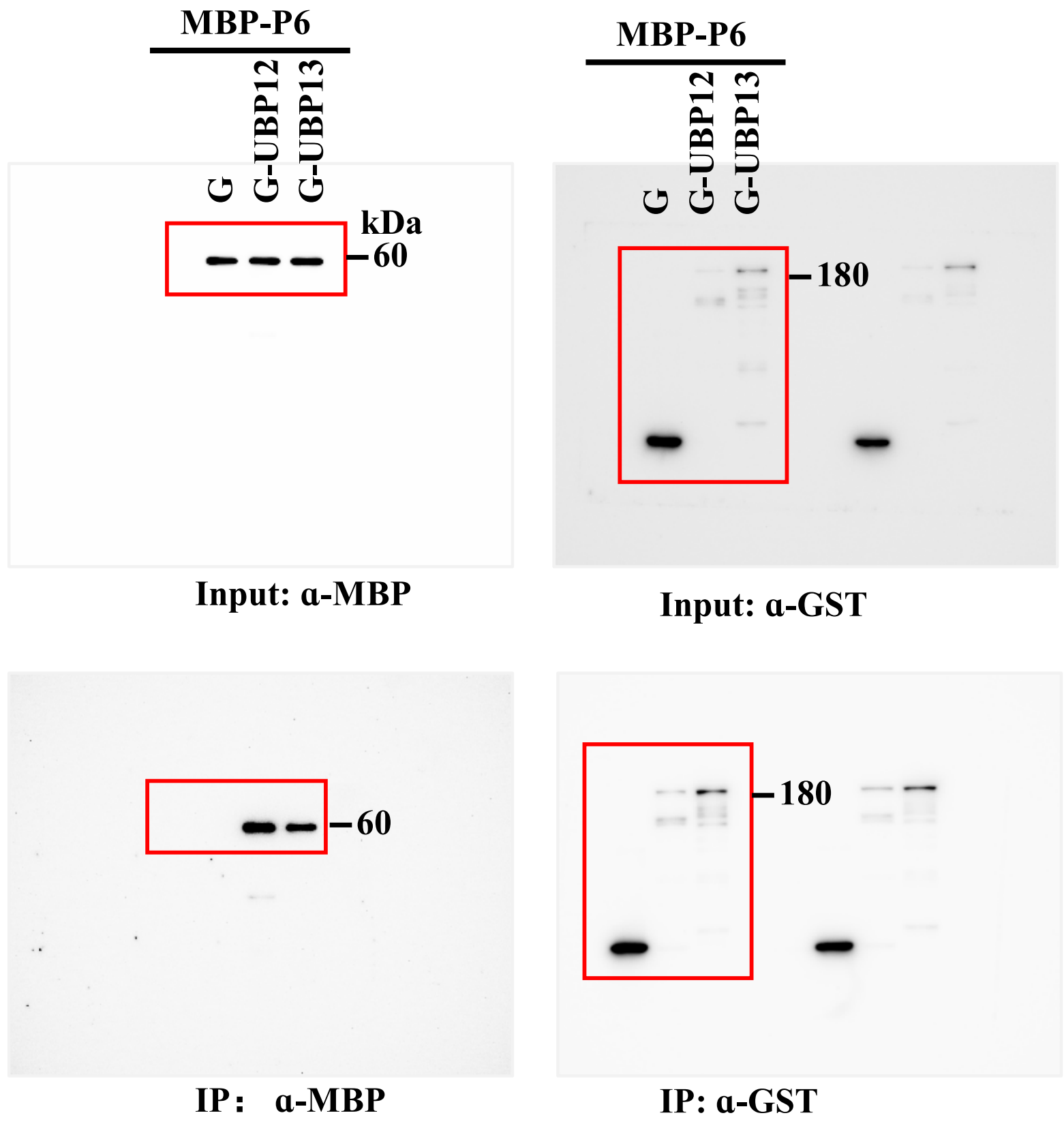

Fig 2C

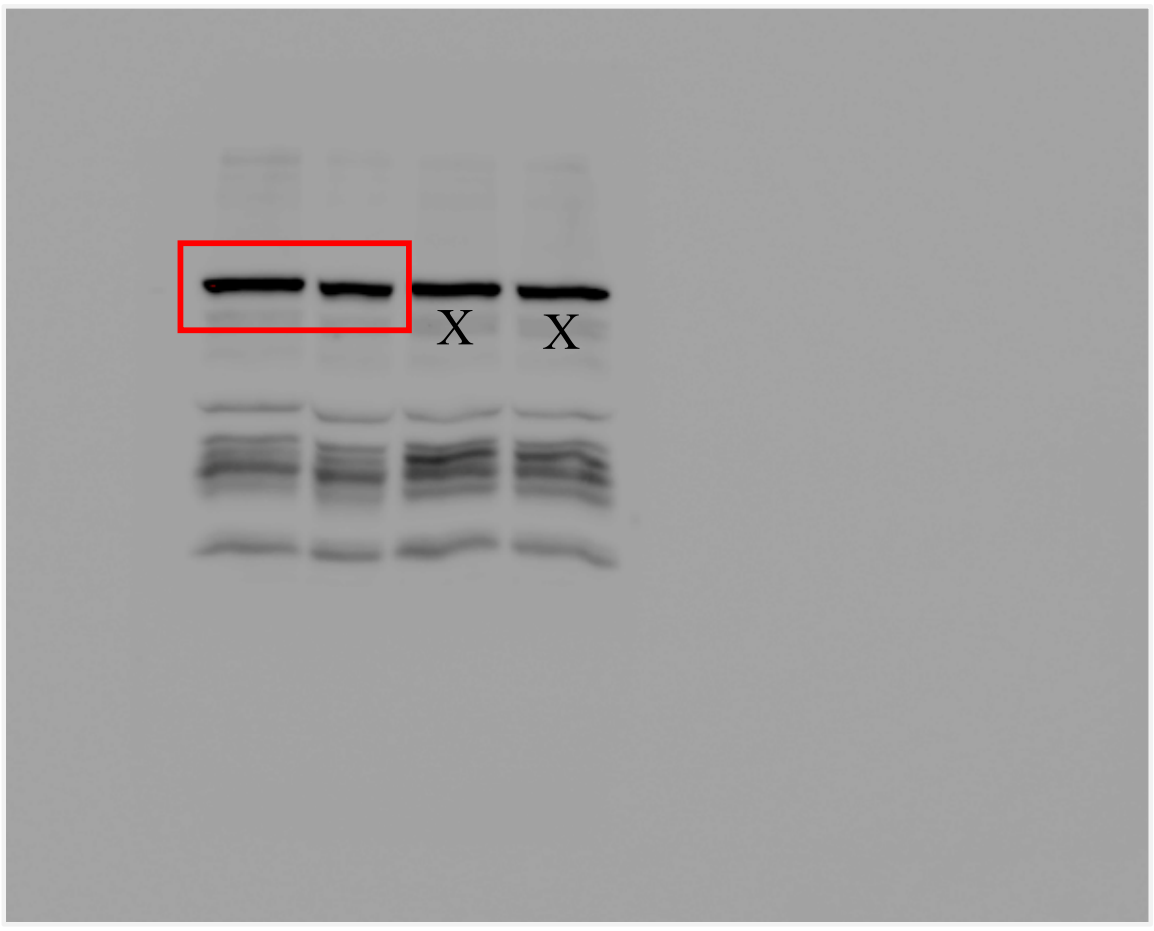

Input: α-GFP

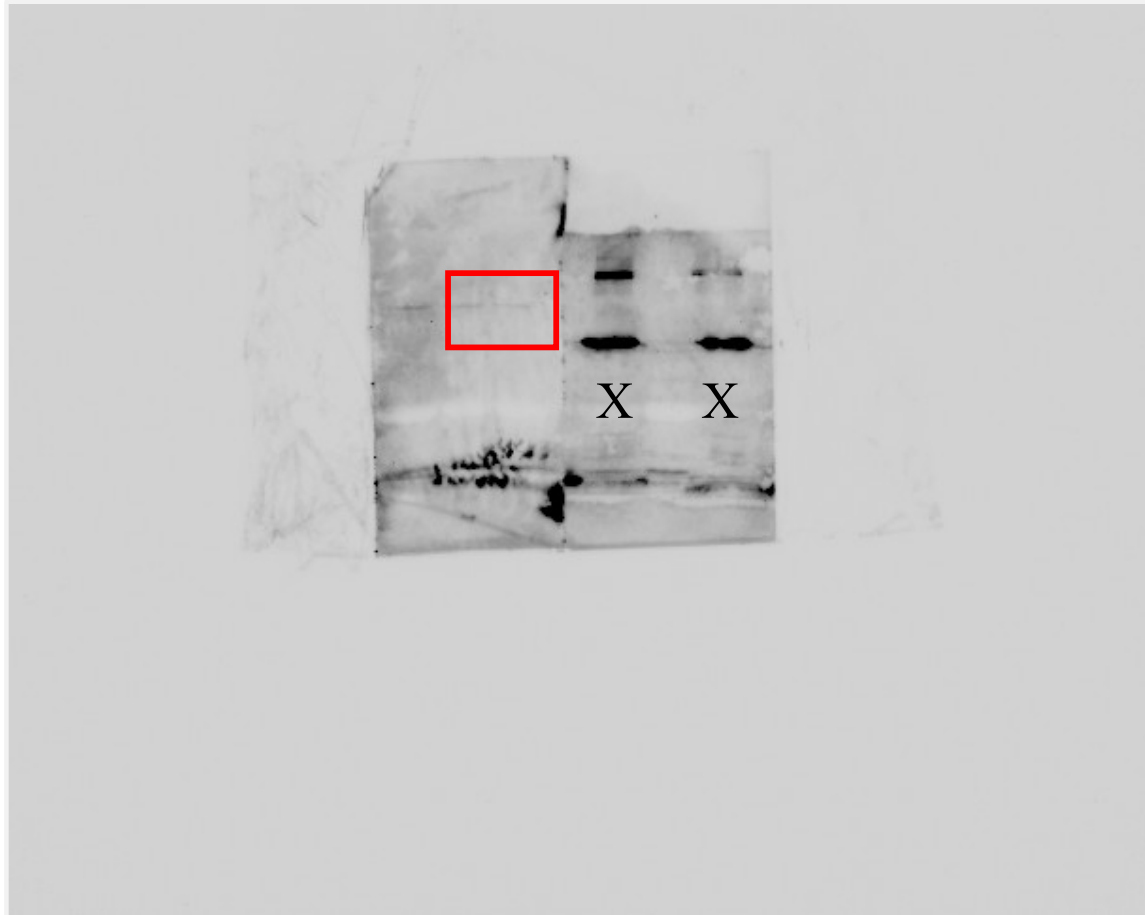

Input: α-FLAG

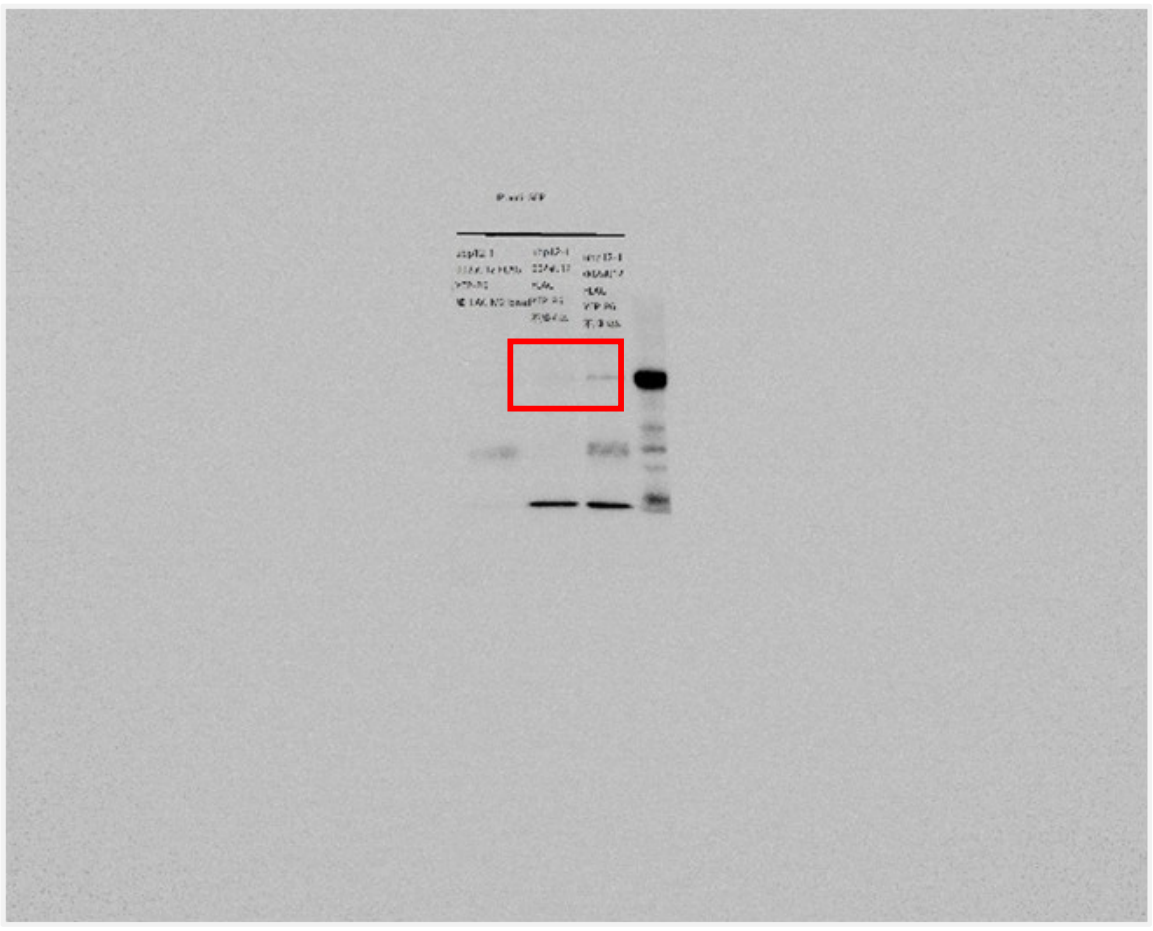

IP: α-GFP

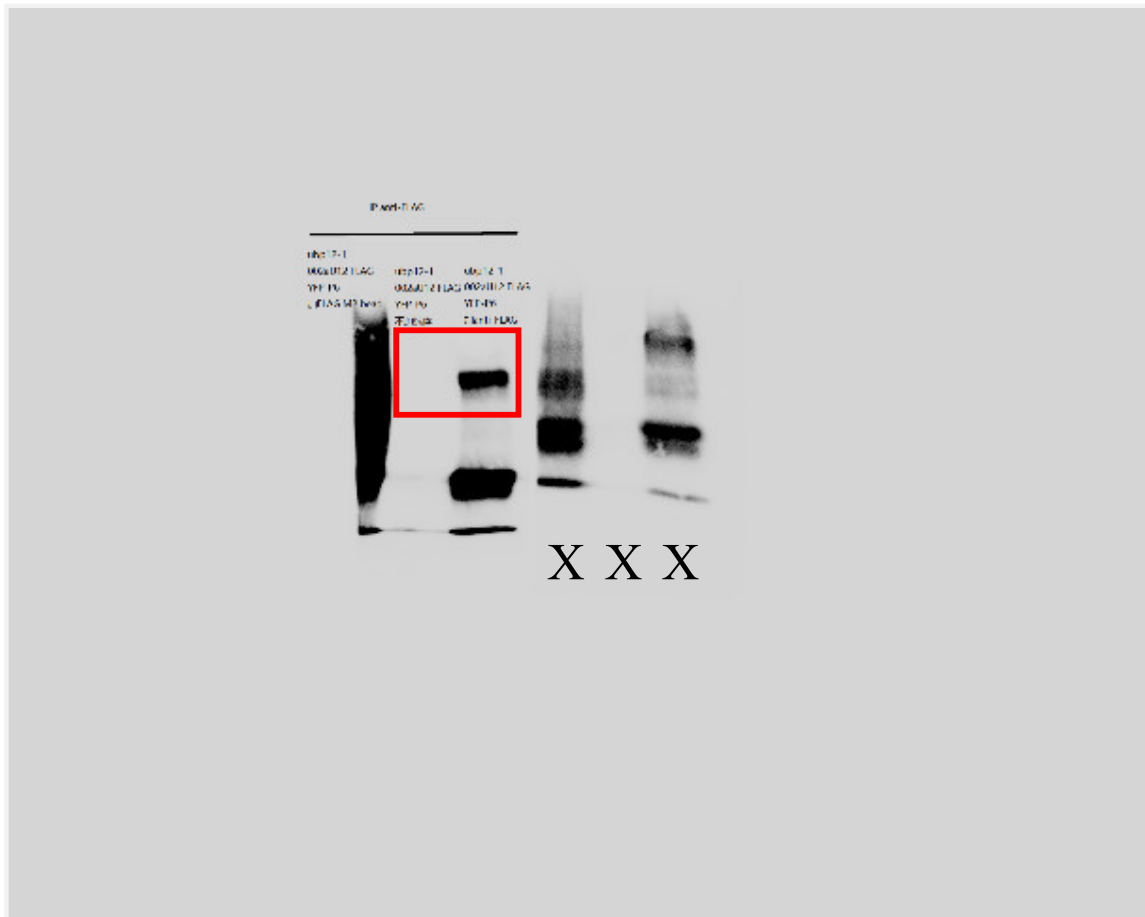

IP: α-FLAG

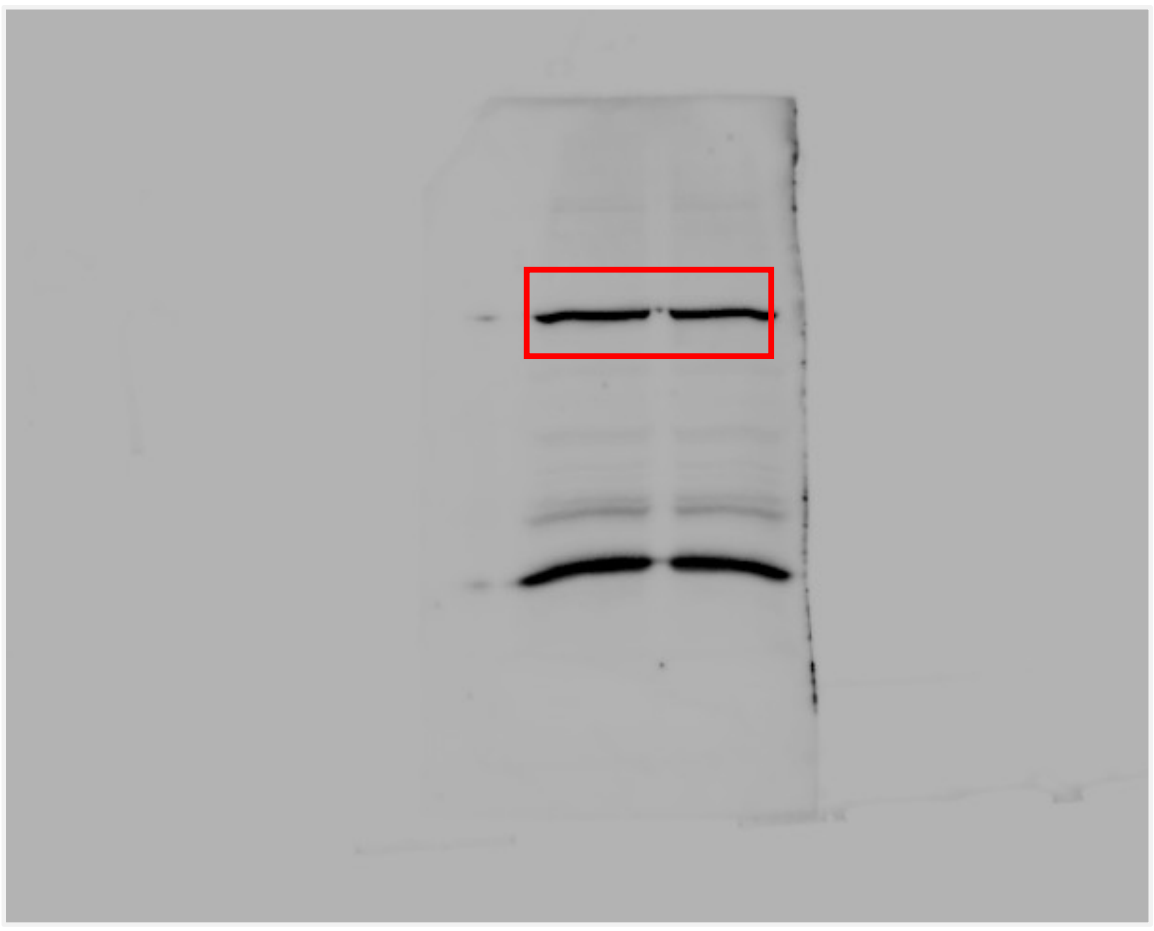

Input: α-GFP

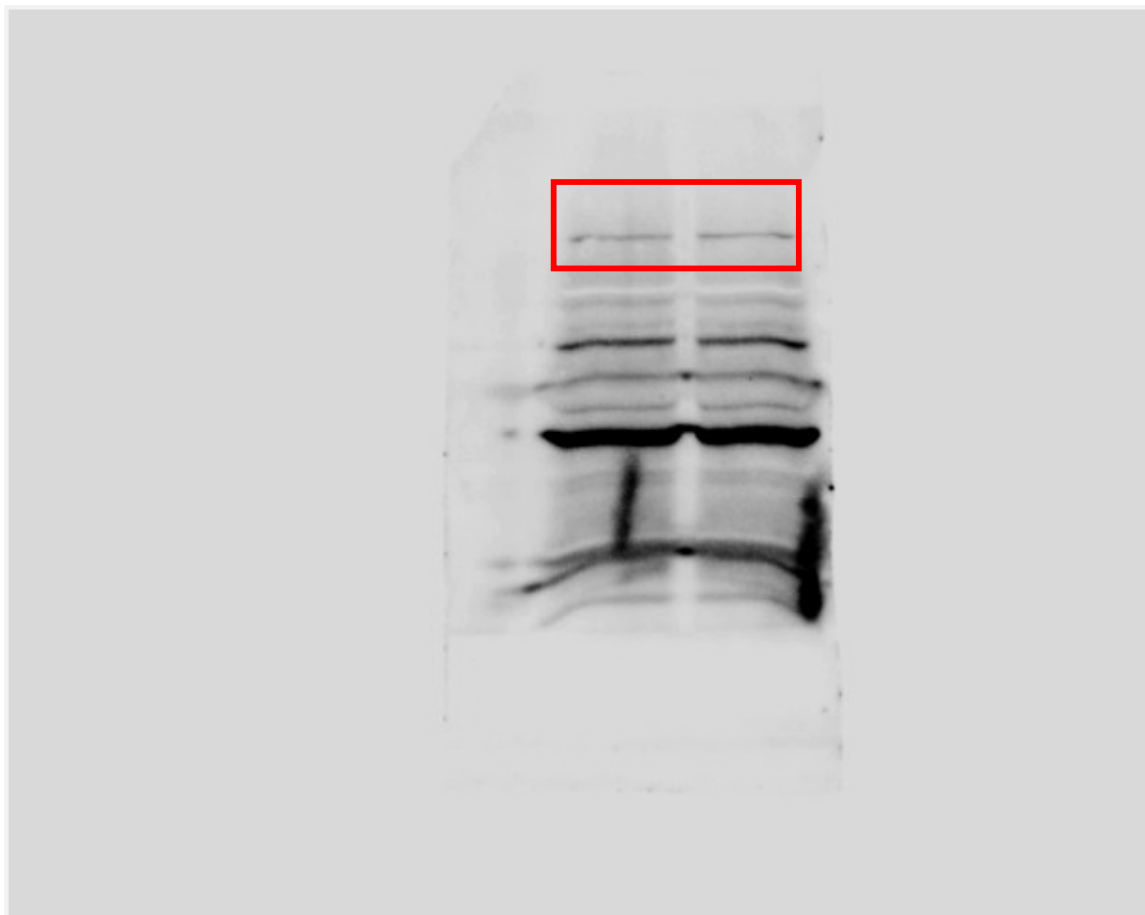

Input: α-FLAG

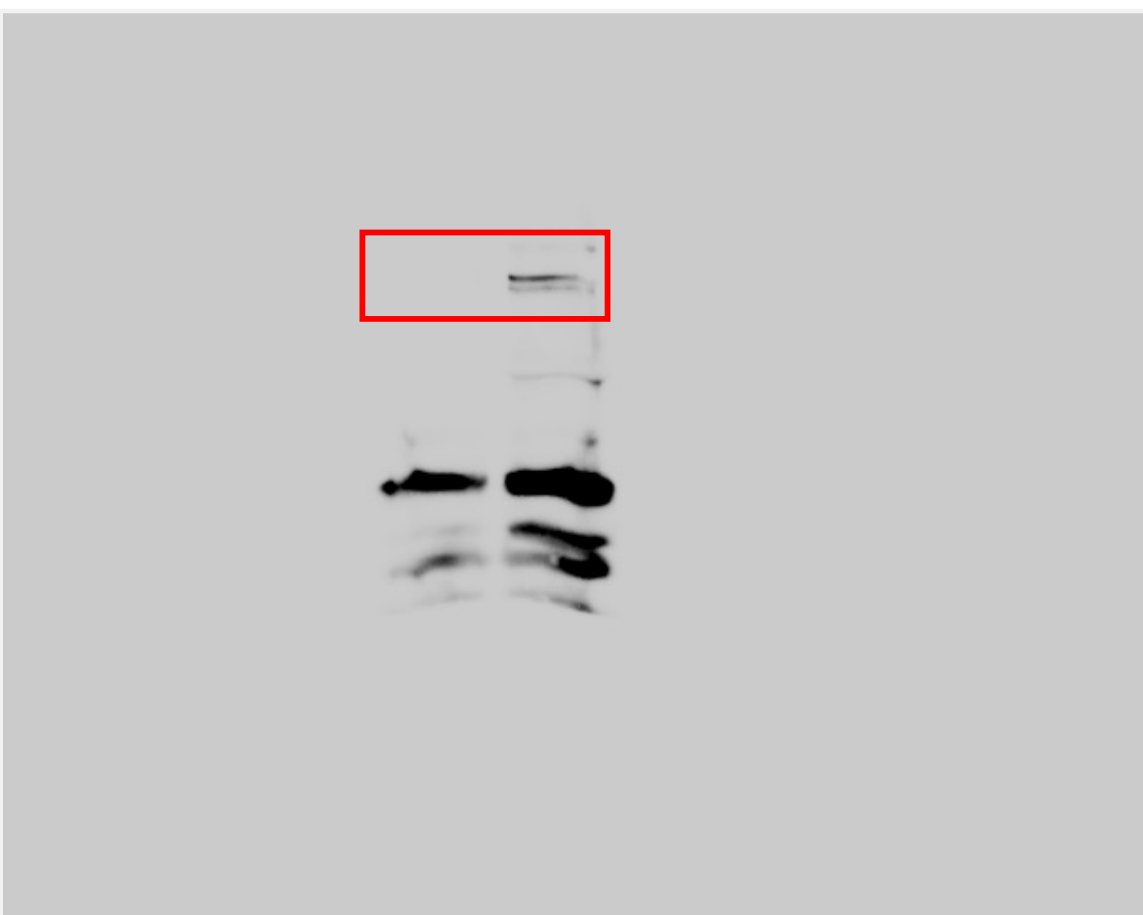

IP: α-GFP

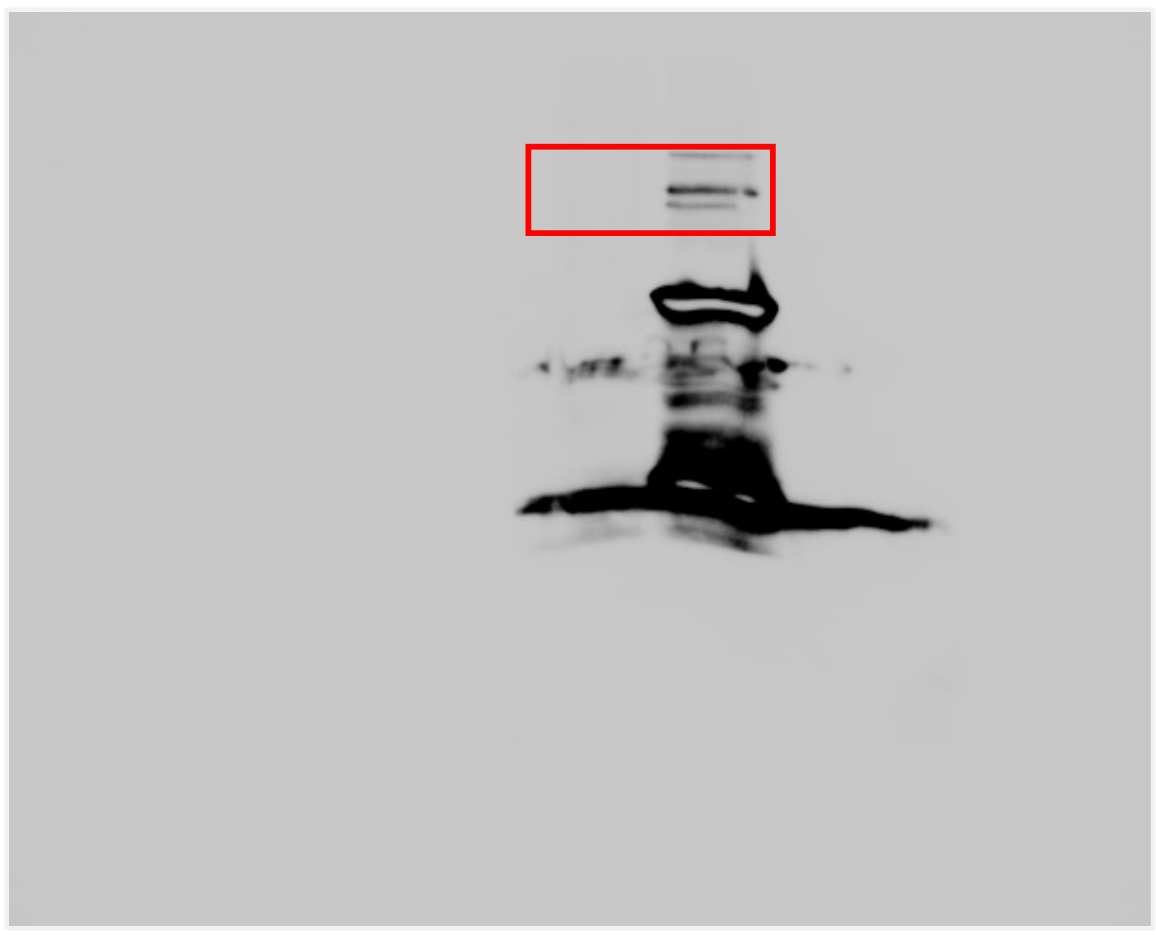

IP: α-FLAG

Fig 4A

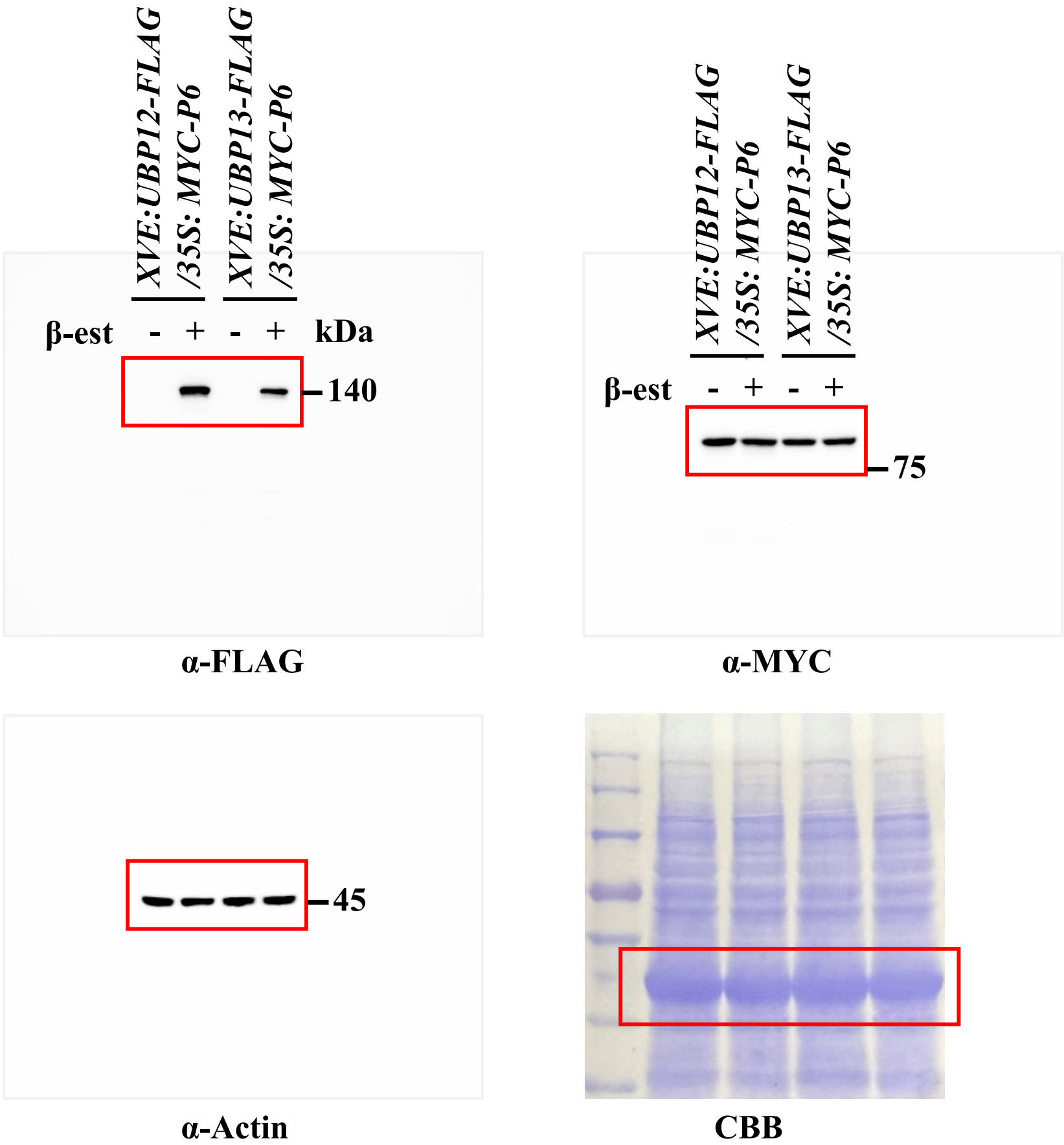
XVE:UBP12-FLAG
/35S: MYC-P6
XVE:UBP13-FLAG
/35S: MYC-P6
β-est
-
+
-
+
kDa
140
α-FLAG
75
α-MYC
45
α-Actin
CBB

Fig 4B

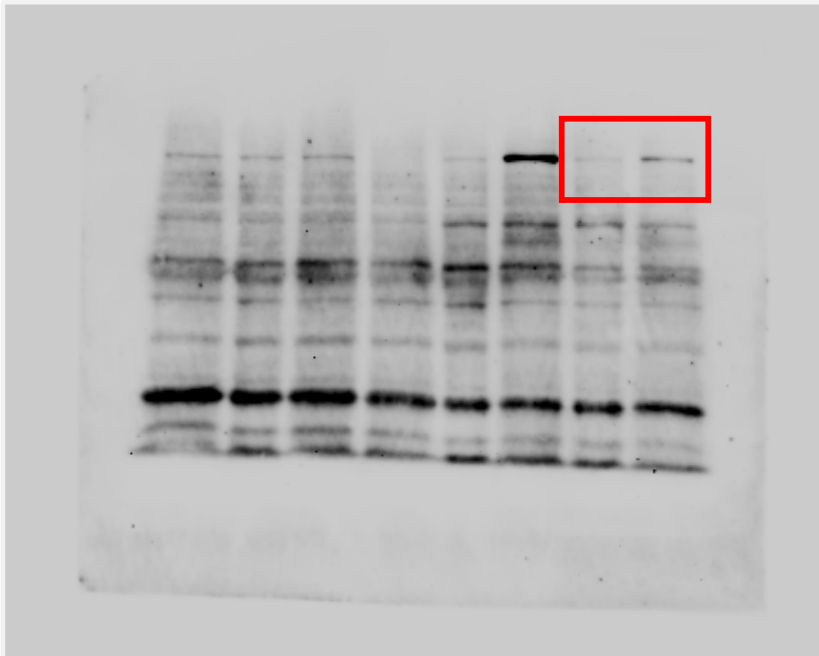

α-FLAG

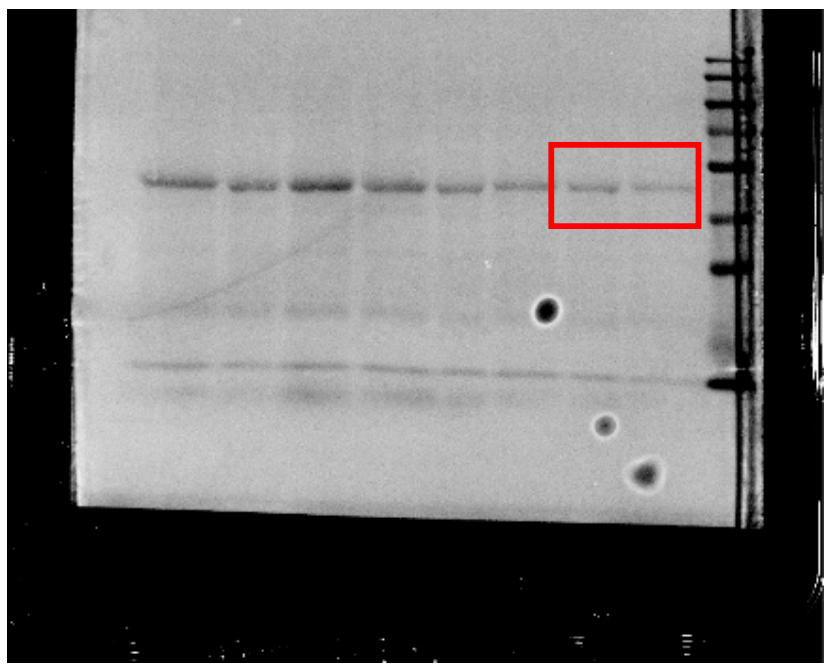

Ponceau *S*

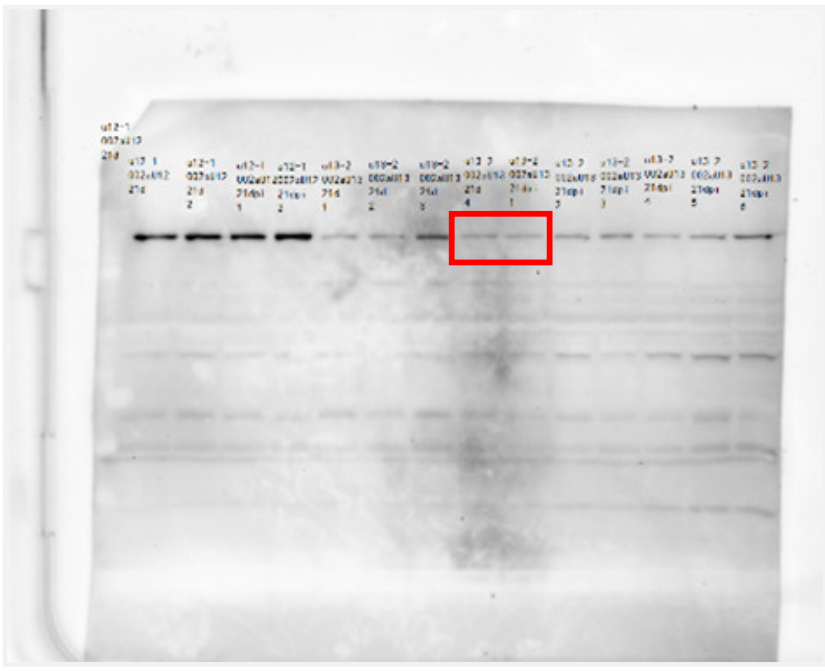

α-FLAG

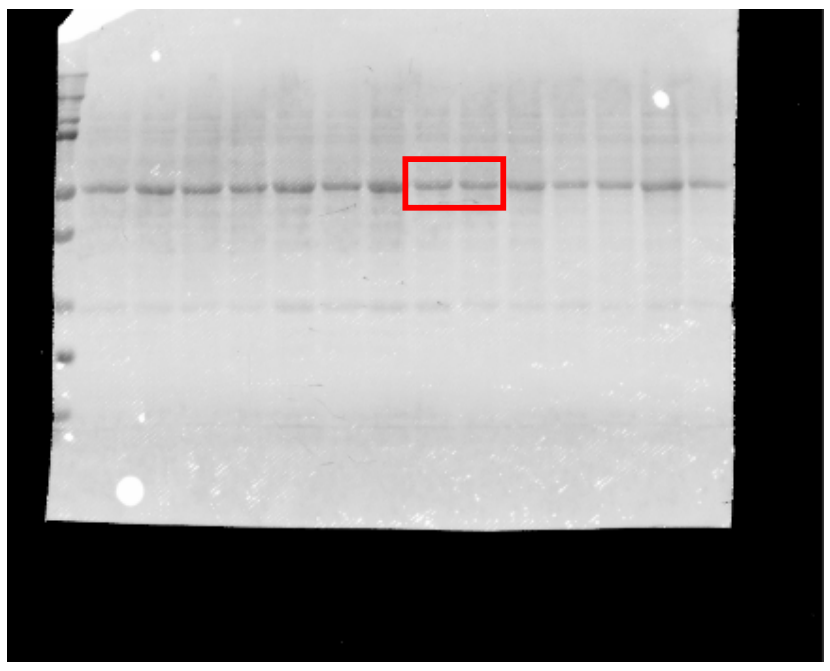

Ponceau *S*

Fig 4C

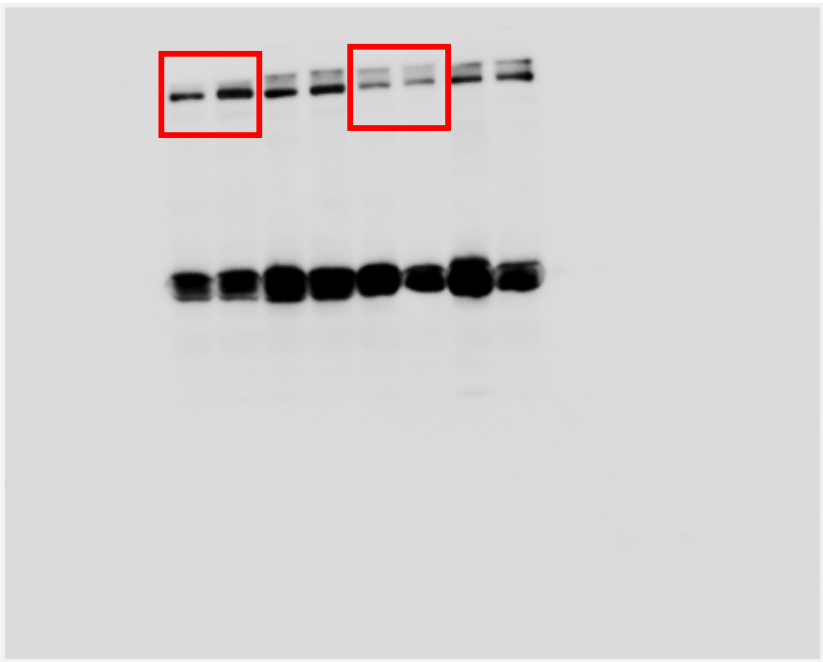

UBP12/13-FLAG

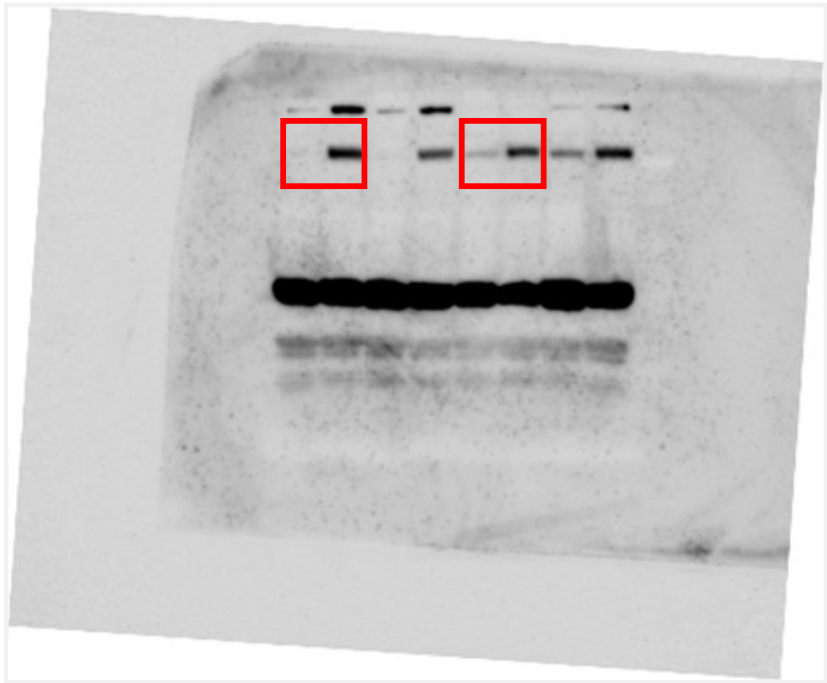

P6-MYC

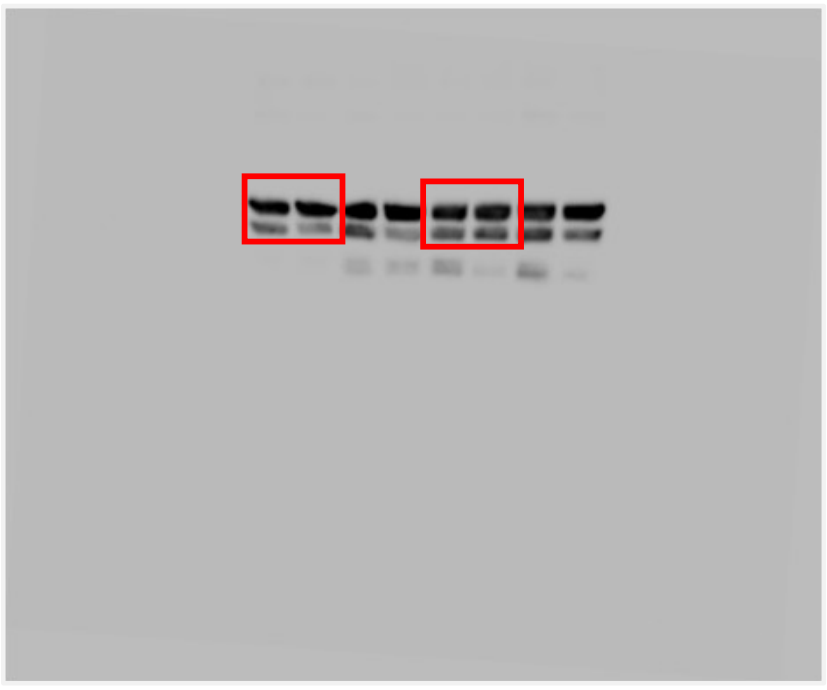

Actin

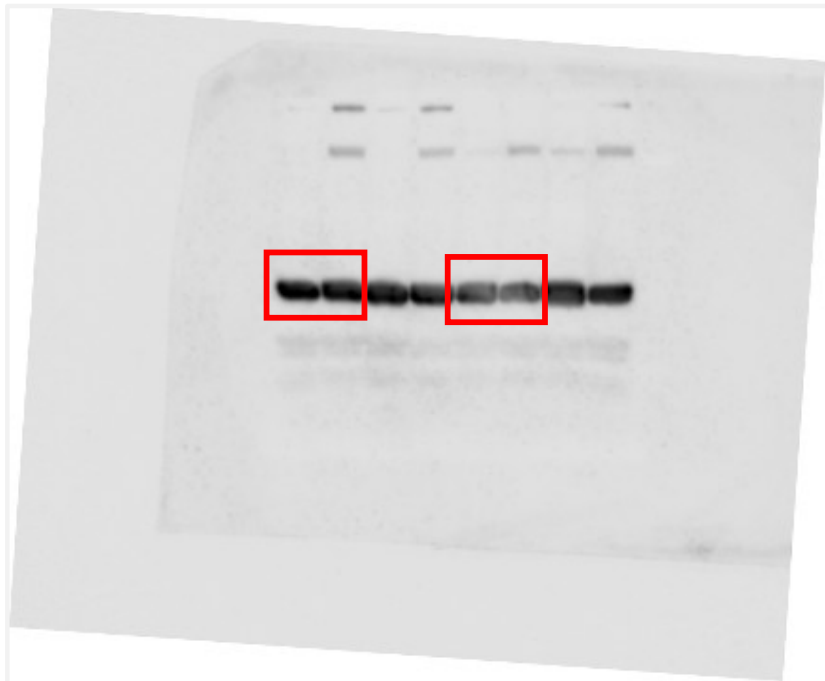

GFP-FLAG

Fig 4D

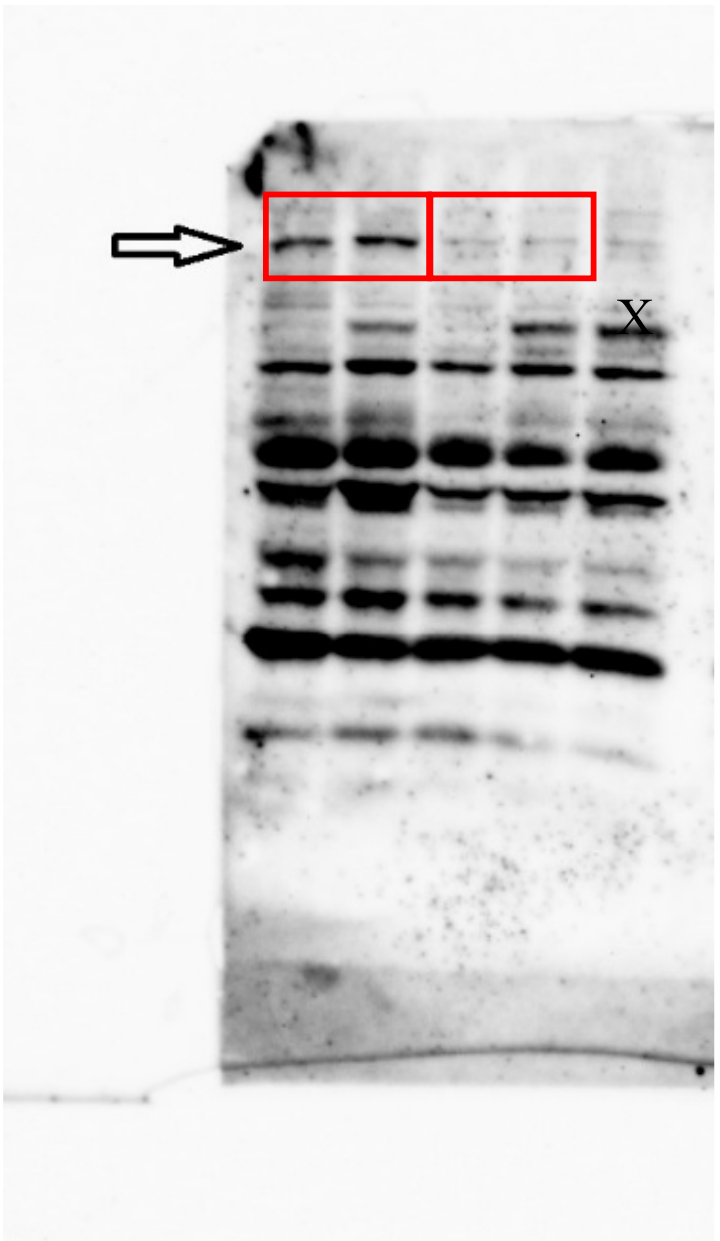

α-FLAG

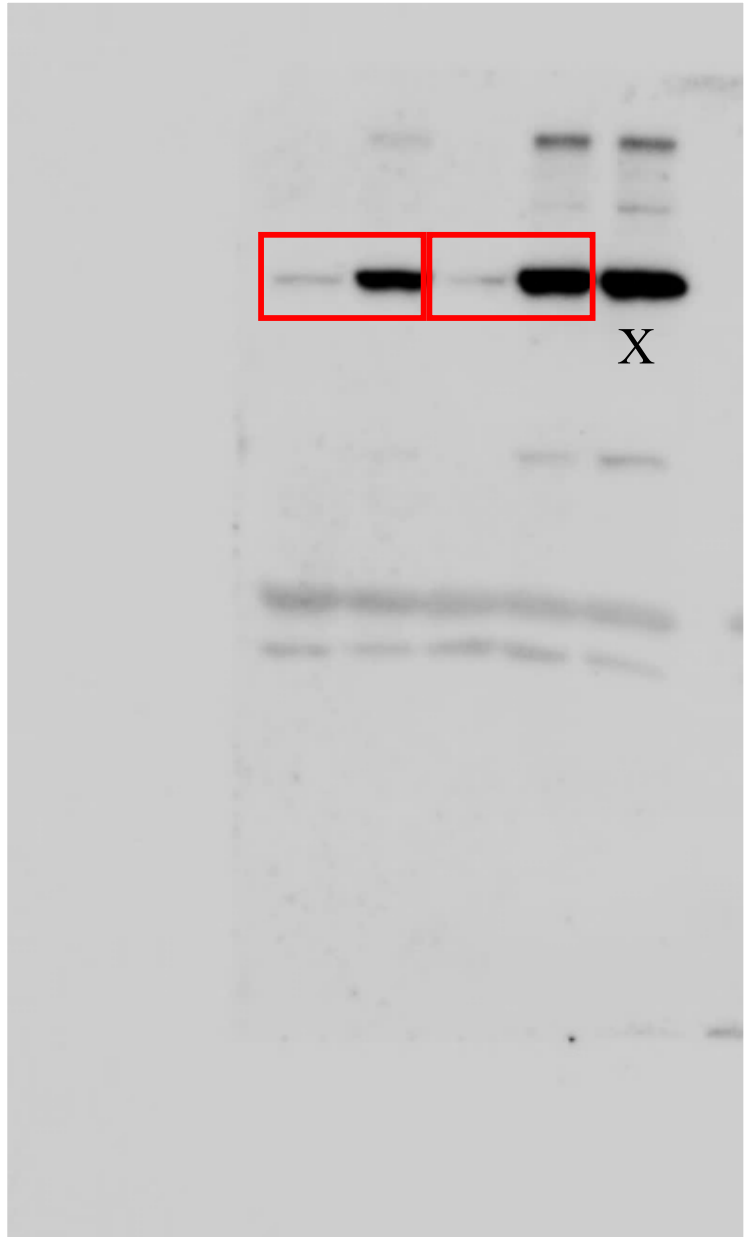

α-MYC

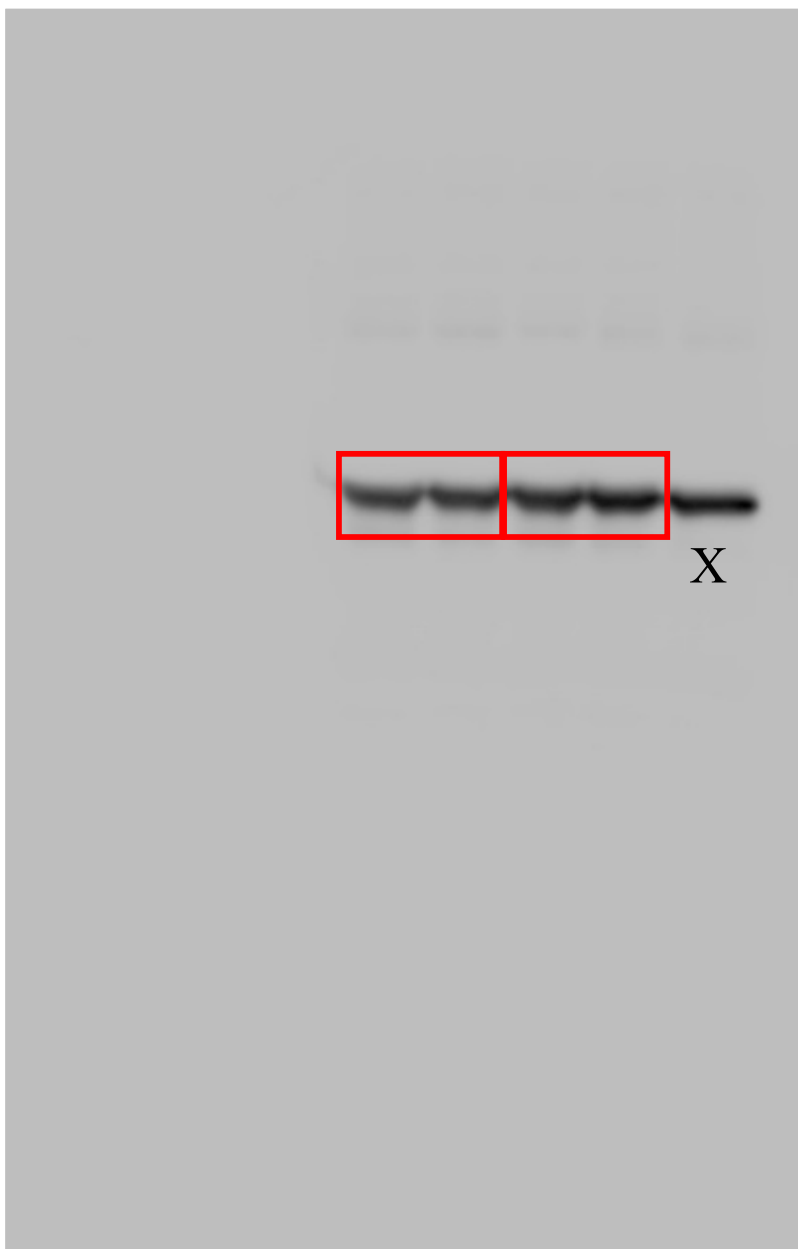

α-Actin

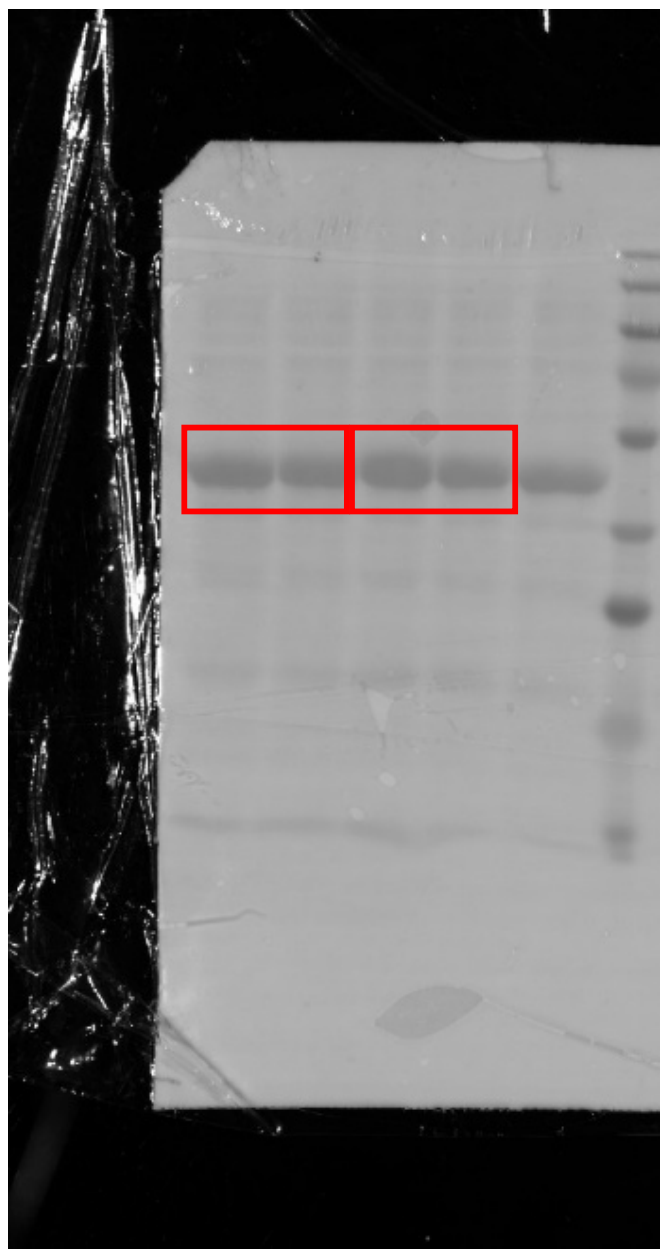

Ponceau *S*

Fig 7A

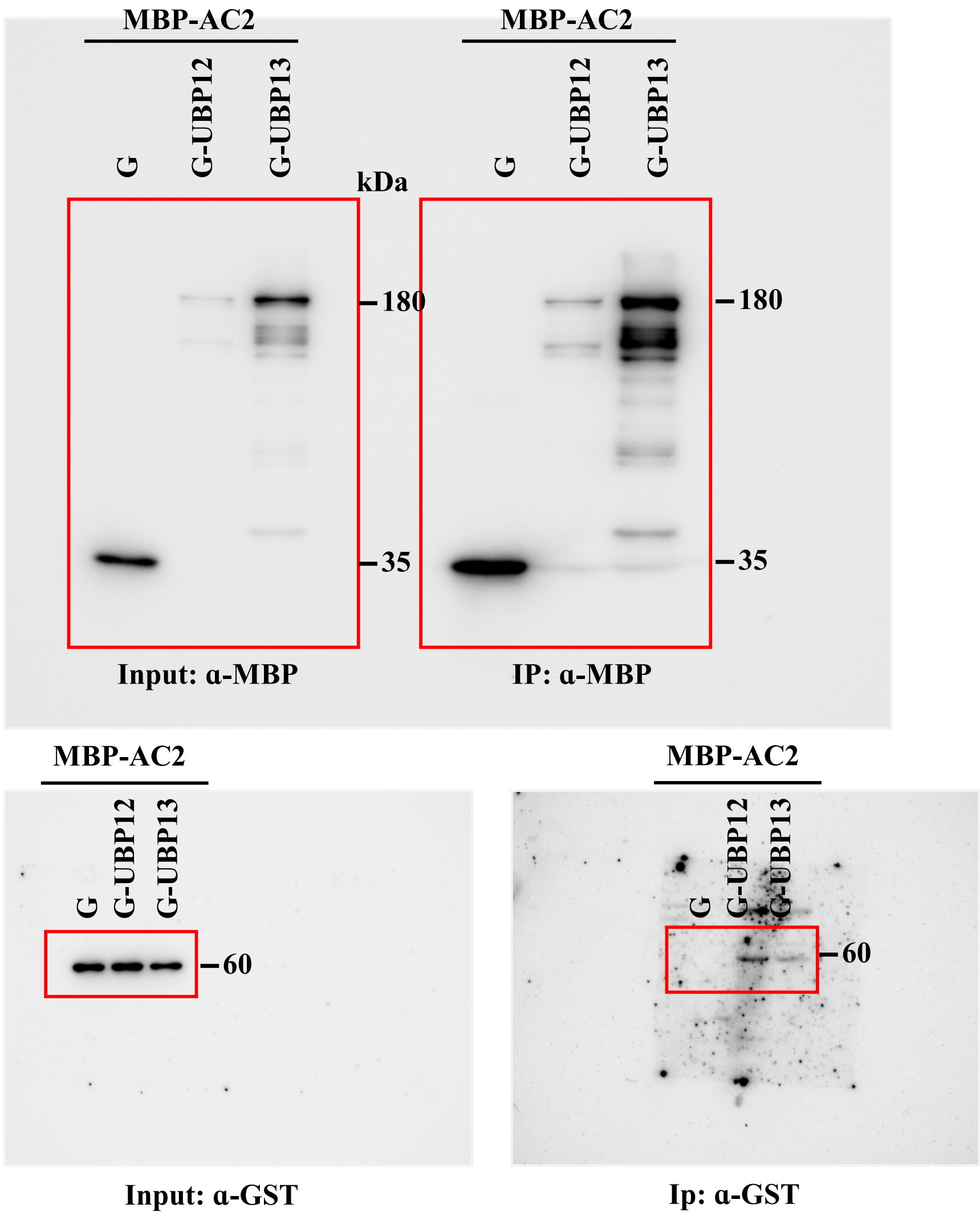
MBP-AC2
G
G-UBP12
G-UBP13
kDa
180
35
Input: α-MBP
MBP-AC2
G
G-UBP12
G-UBP13
180
35
IP: α-MBP
MBP-AC2
G
G-UBP12
G-UBP13
60
Input: α-GST
MBP-AC2
G
G-UBP12
G-UBP13
60
Ip: α-GST

Fig 7B

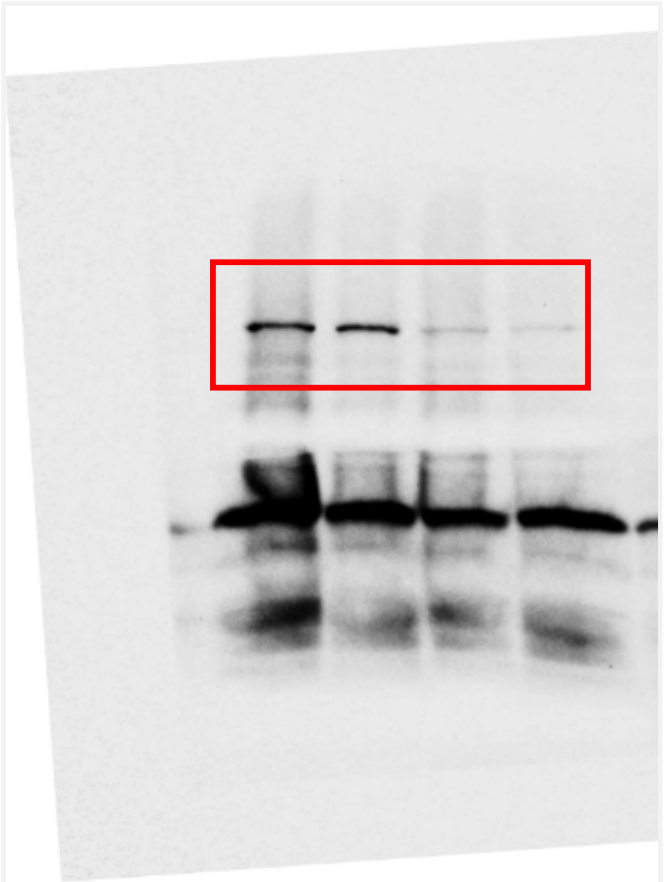

Input: α-FLAG

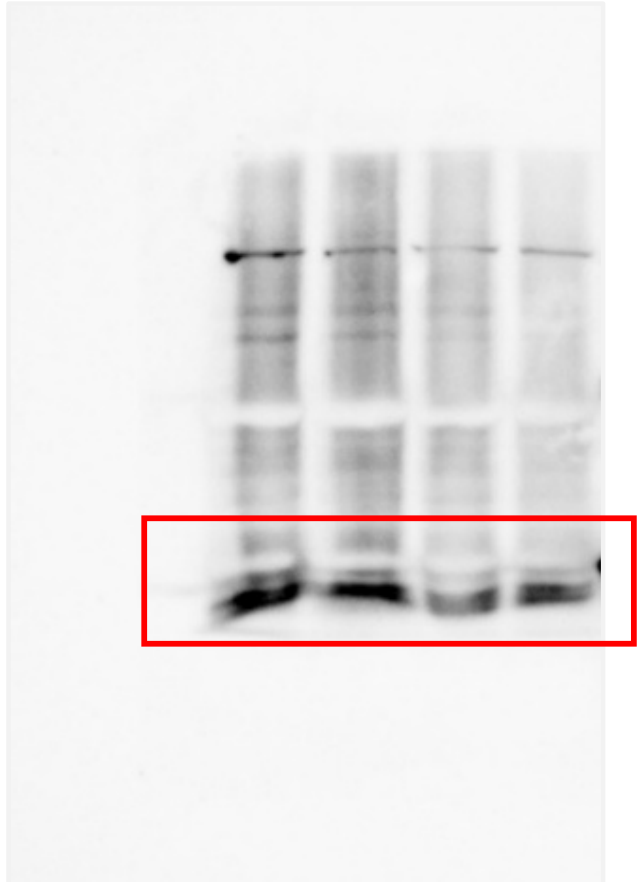

Input: α-MYC

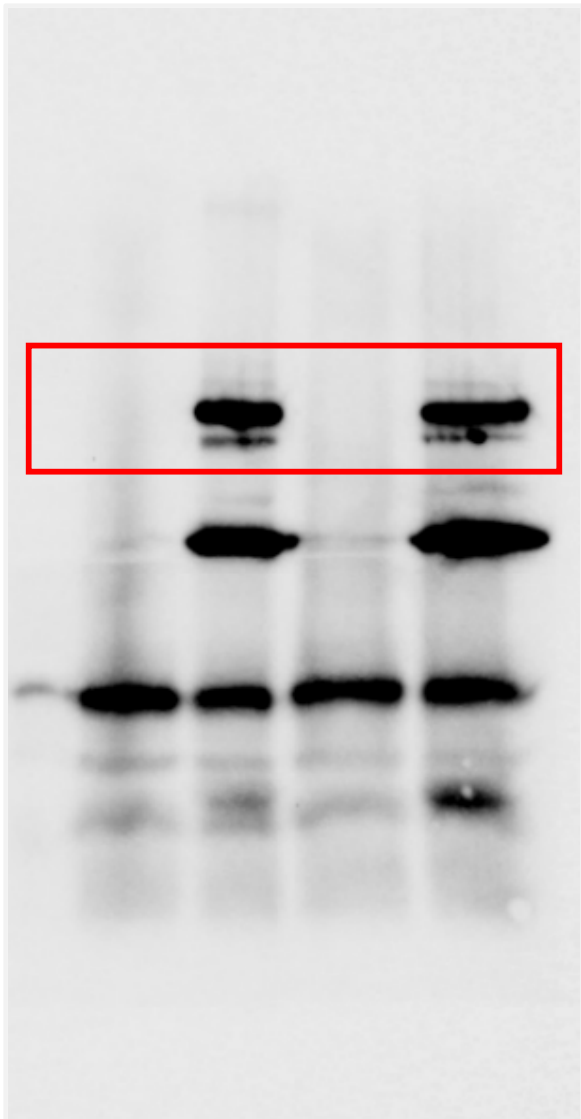

IP: α-FLAG

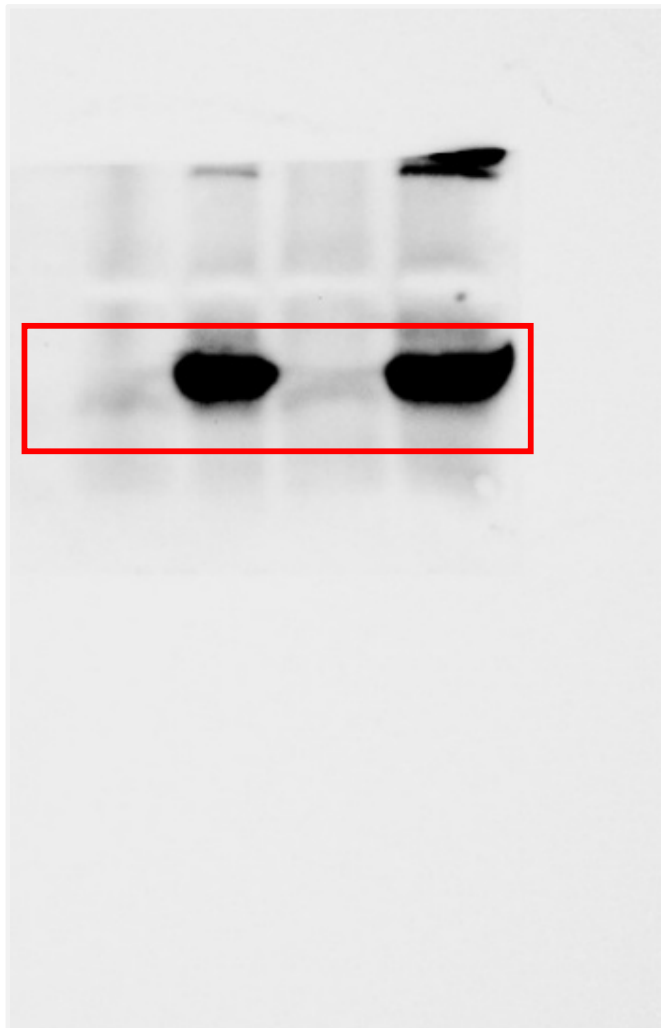

IP: α-MYC

S3 Fig

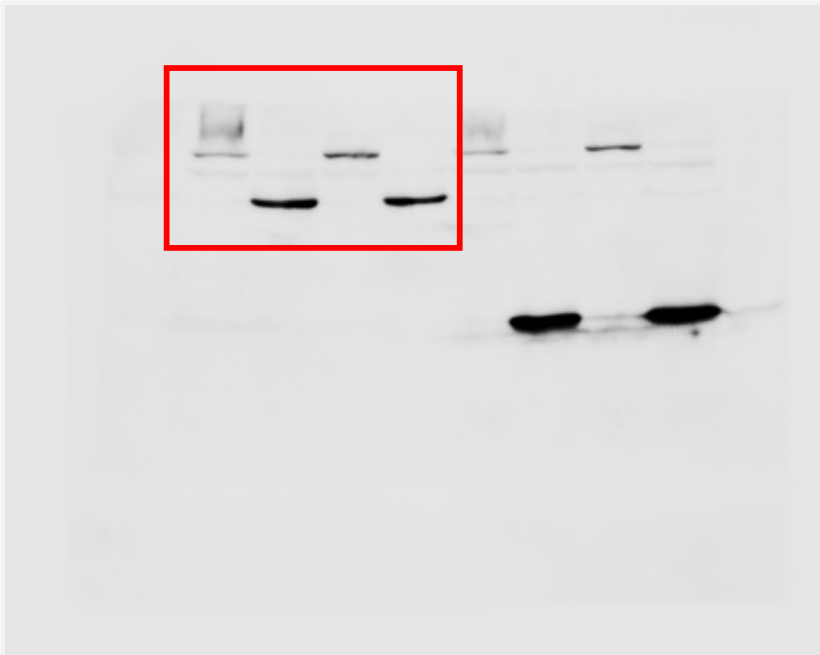

Input: α-FLAG

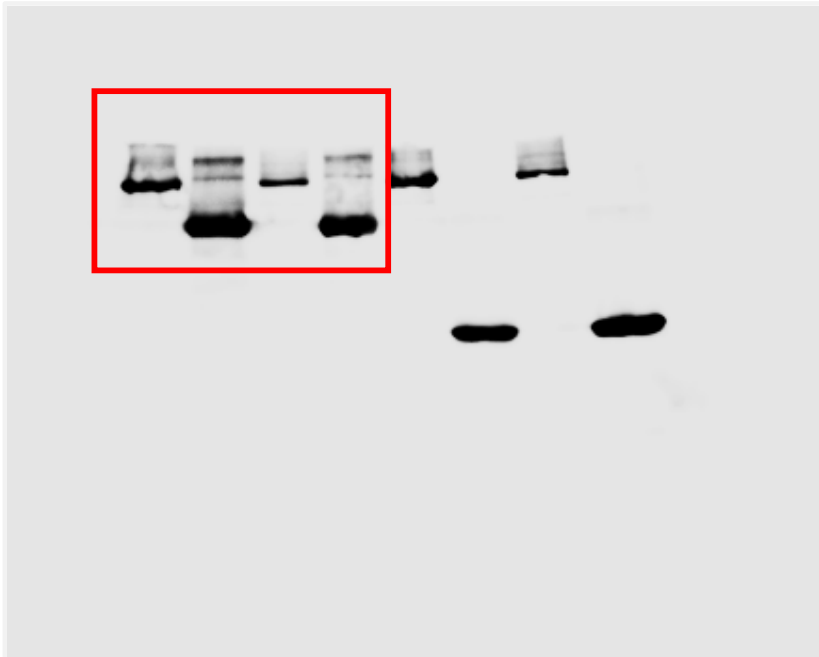

IP: α-FLAG

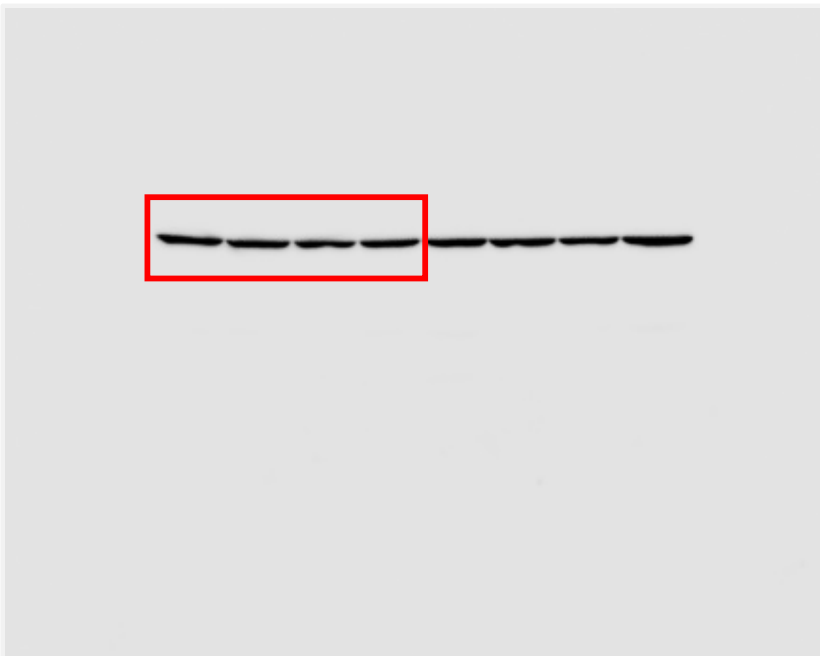

Input: α-MYC

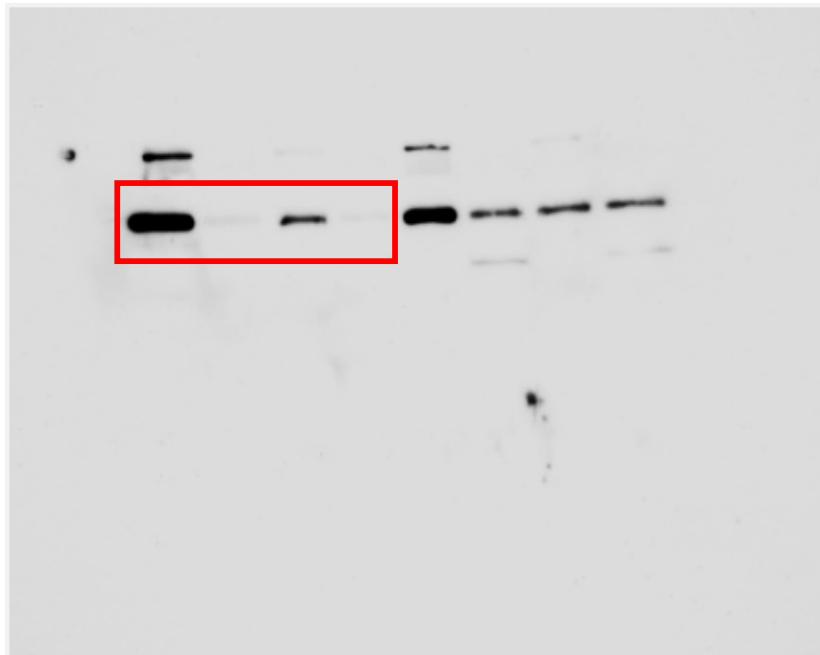

IP: α-MYC

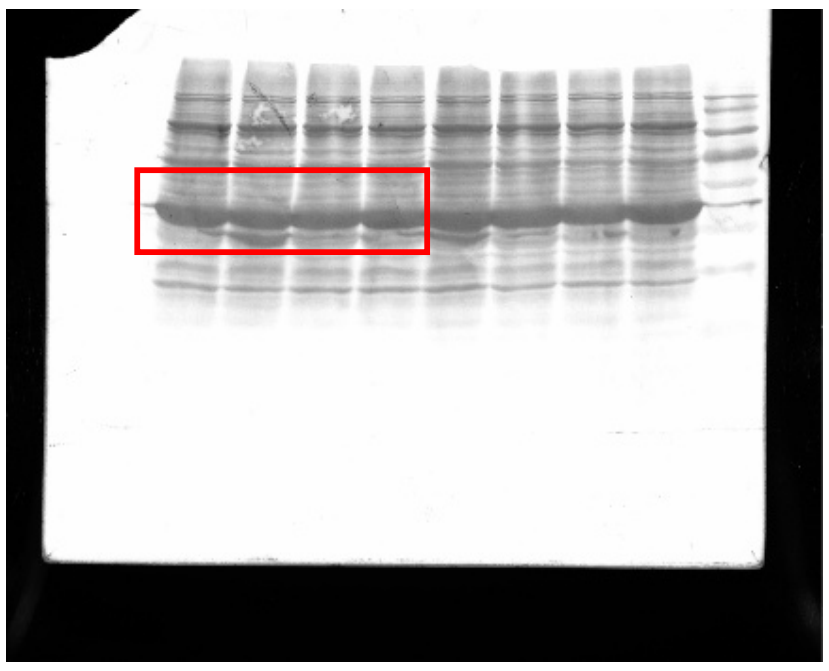

Ponceau *S*

S4A Fig

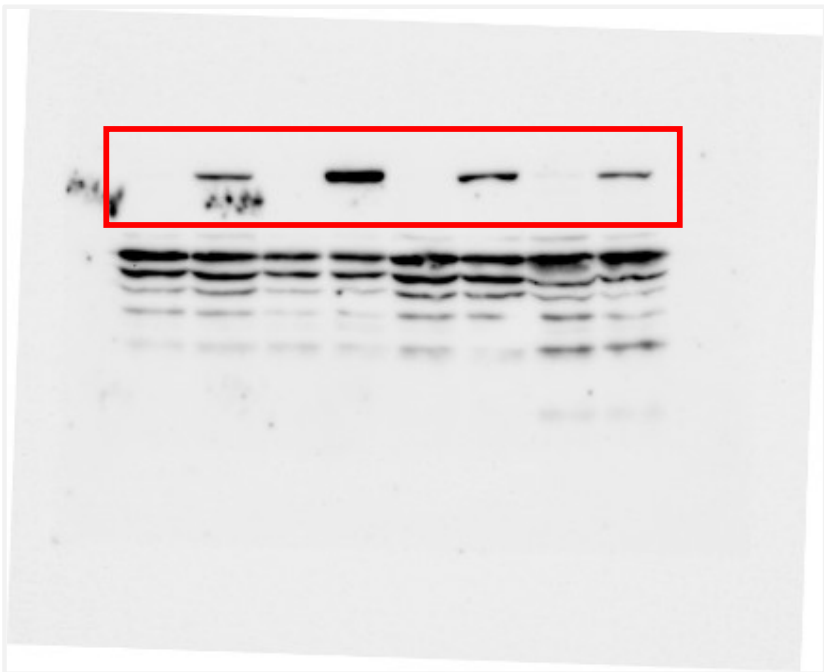

α-FLAG

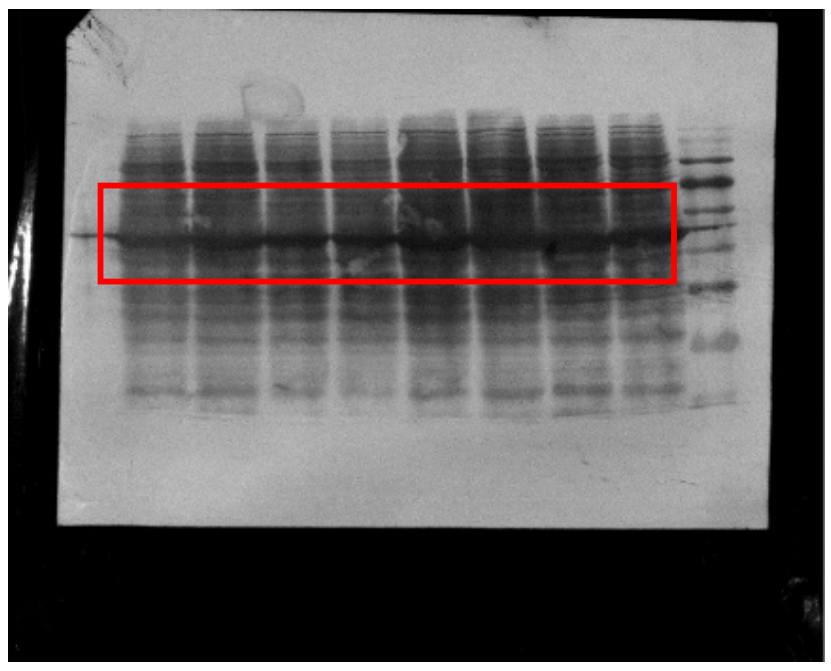

Ponceau *S*

S5 Fig

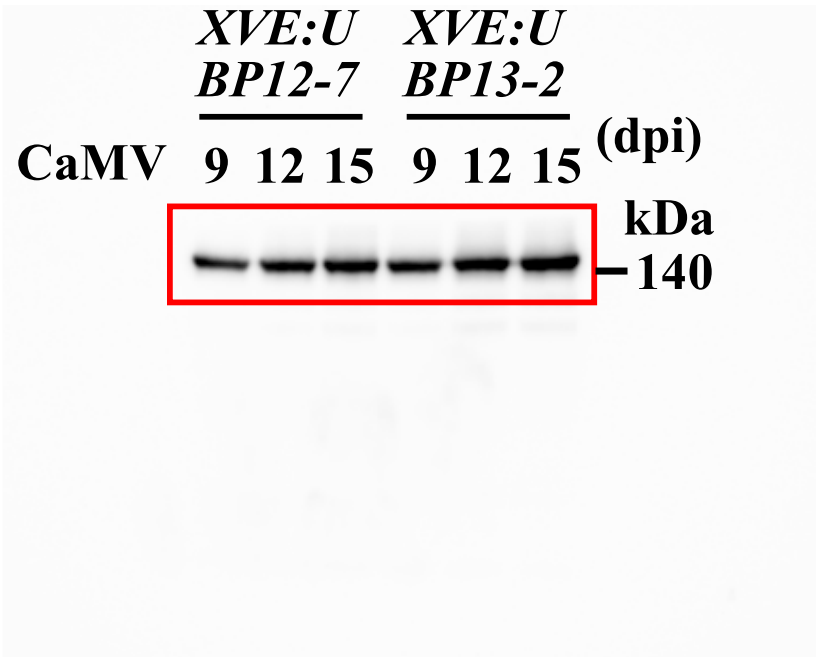

α-FLAG

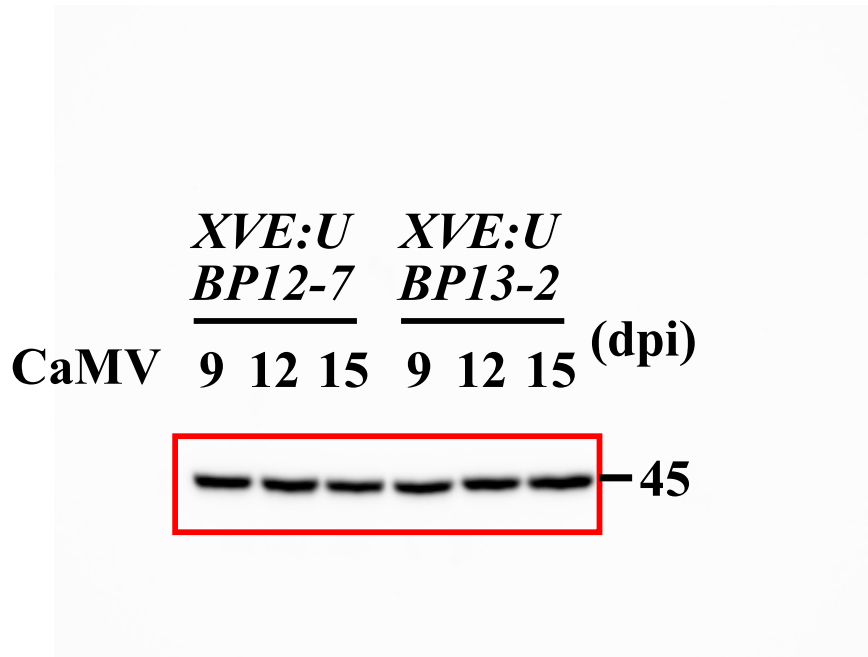

α-Actin

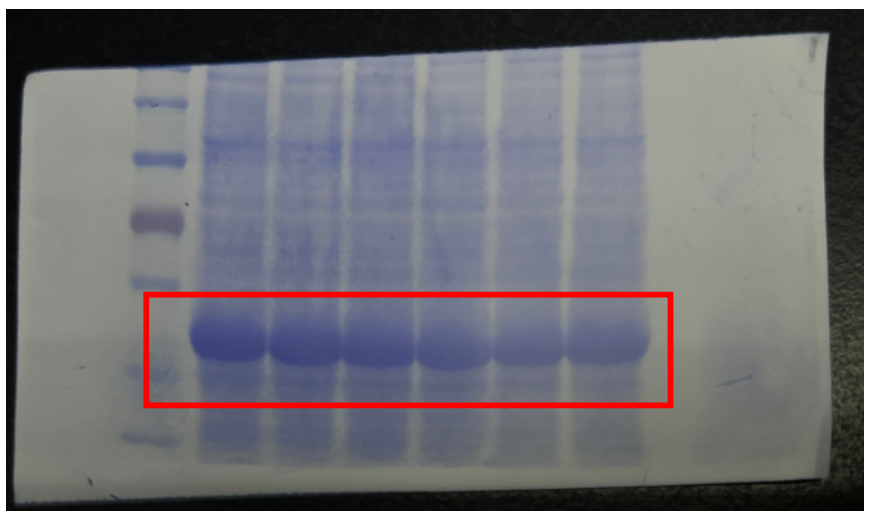

CBB

S6A Fig

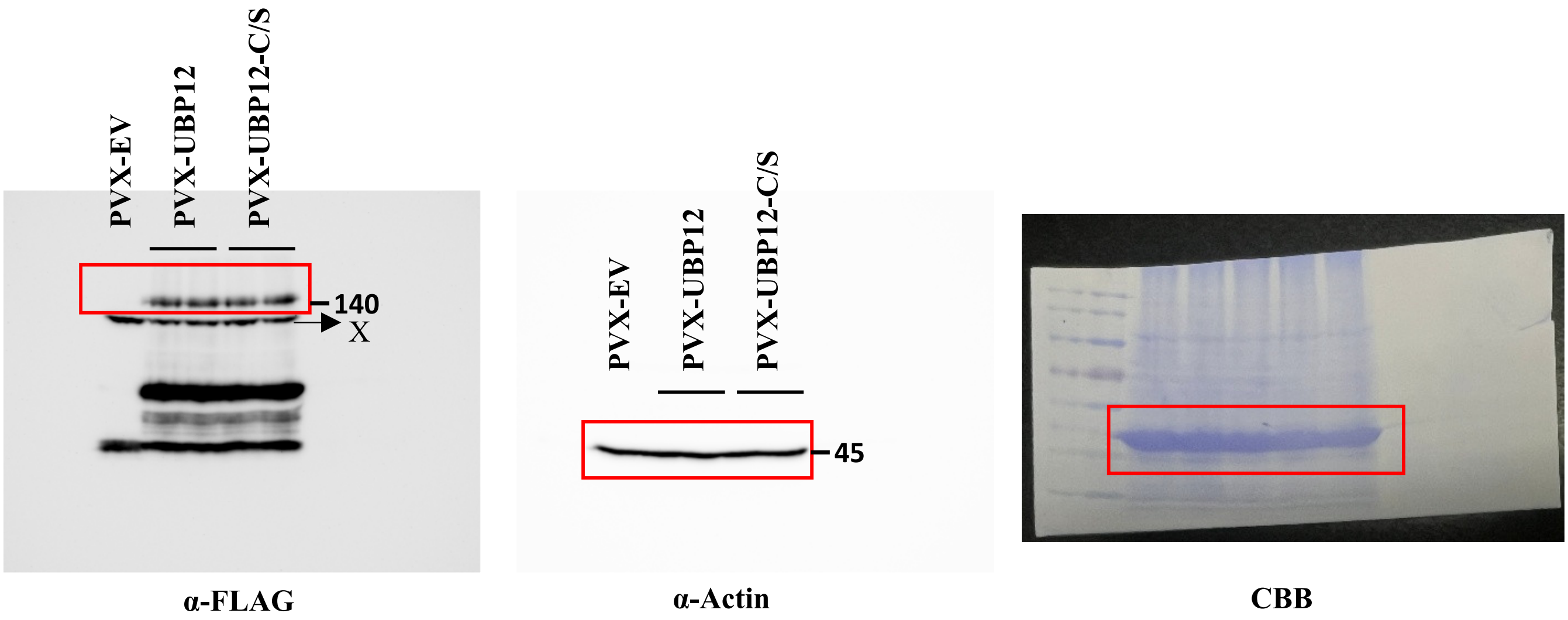

S6C Fig

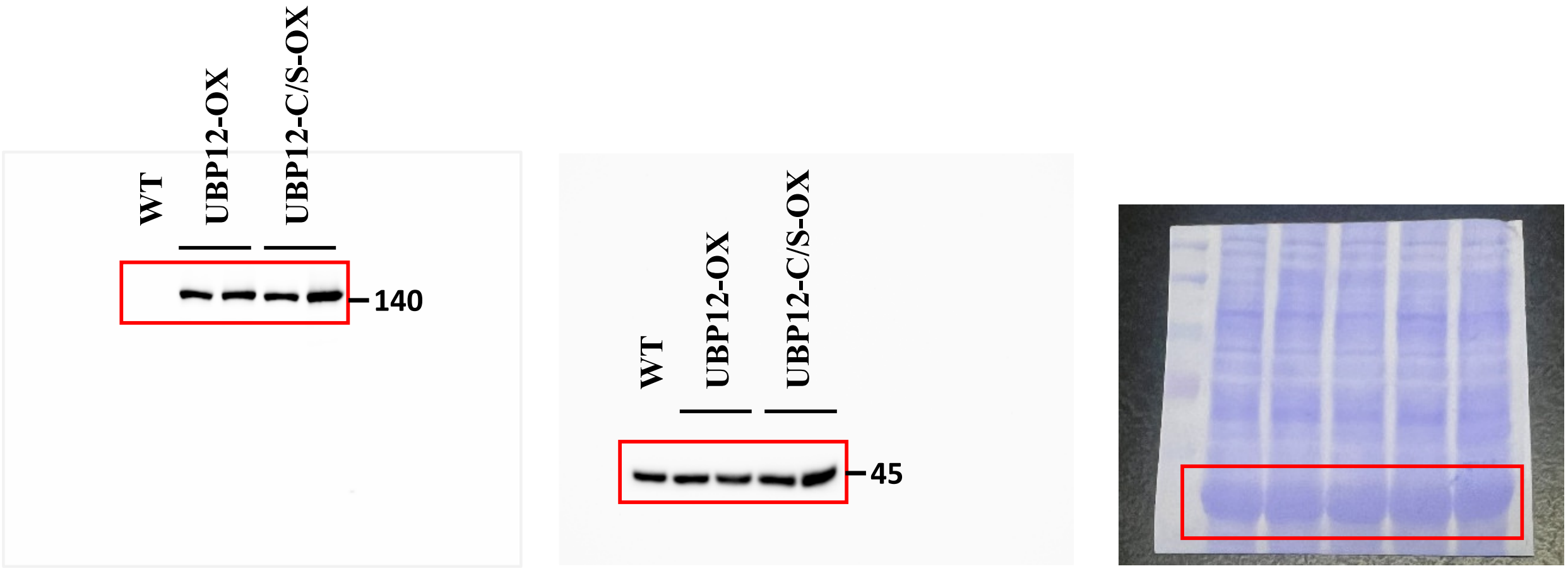

α-FLAG α-FLAG CBB

S7 Fig

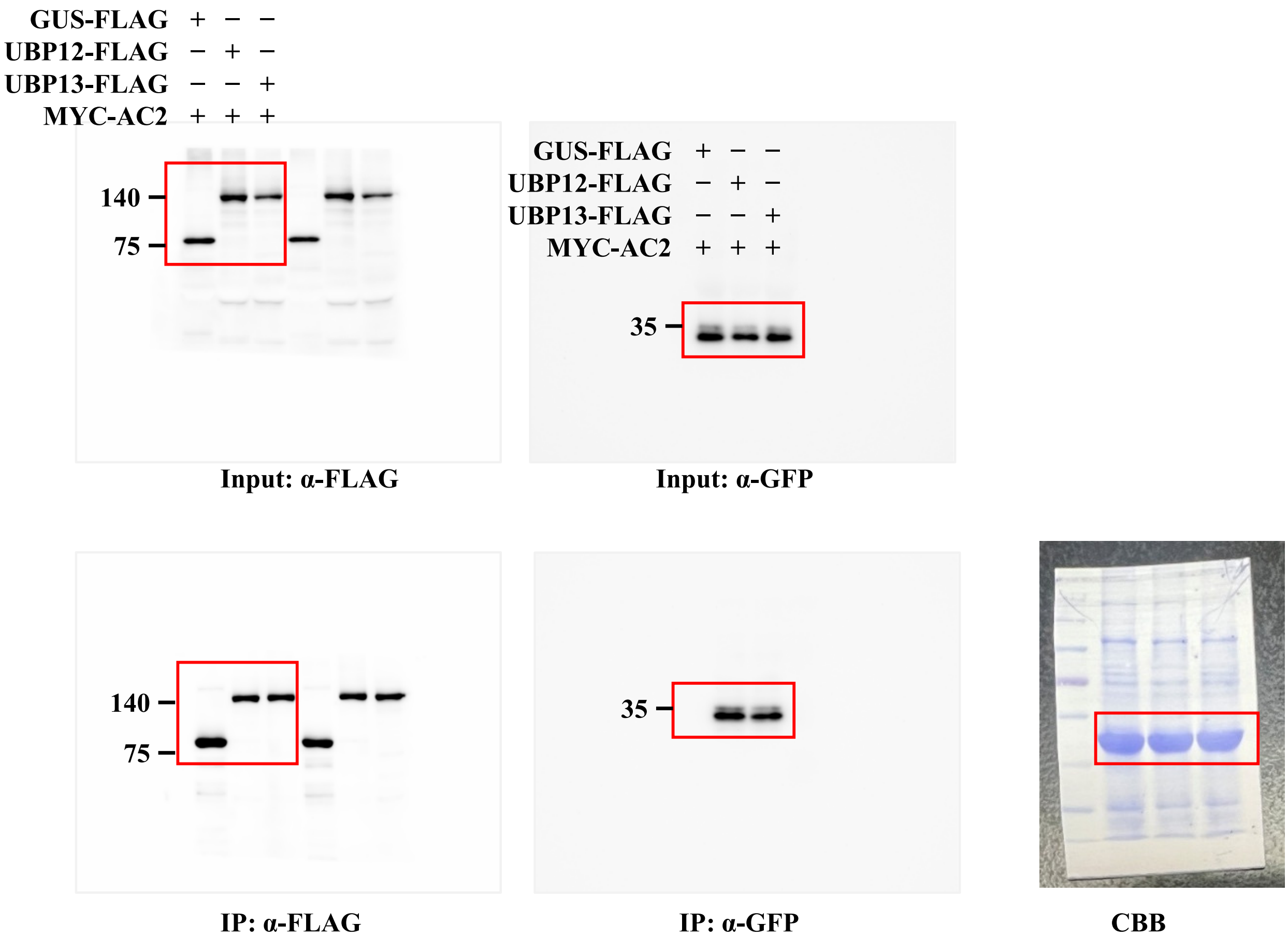
GUS-FLAG + − −
UBP12-FLAG − + −
UBP13-FLAG − − +
MYC-AC2 + + +
140
75
Input: α-FLAG
GUS-FLAG + − −
UBP12-FLAG − + −
UBP13-FLAG − − +
MYC-AC2 + + +
35
Input: α-GFP
140
75
IP: α-FLAG
35
IP: α-GFP
CBB
